# *Helicobacter pylori*‐Induced Persistent IGF2BP1 Activation Promotes Ferroptosis Resistance in Gastric Tumorigenesis

**DOI:** 10.1002/advs.77677

**Published:** 2026-09-11

**Authors:** Jing Ning, Xin Guan, Xinyu Hao, Jing Zhang, Deyao Li, Ying Xiong, Wenlin Zhang, Xiurui Han, Yanfei Lang, Meiling Zhou, Mengjie Yang, Zhi Huang, Tinghui Qu, Ming Zu, Qiao Meng, Weiwei Fu, Fengmin Lu, Jing Zhang, Xiangmei Chen, Tong Liu, Shigang Ding

**Affiliations:** ^1^ Department of Gastroenterology Beijing Key Laboratory for Helicobacter Pylori Infection and Upper Gastrointestinal Diseases (BZ0371) Peking University Third Hospital Beijing China; ^2^ Department of Microbiology and Infectious Disease Center School of Basic Medical Sciences Peking University Health Science Center Beijing China; ^3^ Institute of Medical Innovation and Research Peking University Third Hospital Beijing China

**Keywords:** epitranscriptomic regulation, ferroptosis resistance, gastric cancer, *Helicobacter pylori*, insulin‐like growth factor 2 mRNA‐binding protein 1 (IGF2BP1)

## Abstract

*Helicobacter pylori* (*H. pylori*) infection is a major risk factor for gastric cancer, yet how a transient bacterial insult induces durable oncogenic reprogramming in gastric epithelial cells remains incompletely understood. Here, we identify insulin‐like growth factor 2 mRNA‐binding protein 1 (IGF2BP1) as a key mediator of sustained ferroptosis resistance during gastric tumorigenesis. We show that *H. pylori* infection induces IGF2BP1 expression in gastric epithelial cells, murine models, and human gastric tissues, and that IGF2BP1 upregulation can persist following bacterial eradication, consistent with a potential “hit‐and‐run”‐like mode of epithelial reprogramming. Functionally, sustained IGF2BP1 expression promotes malignant cell survival and tumor growth by suppressing oxidative stress and ferroptosis. Mechanistically, IGF2BP1 functions as an *N^6^*‐methyladenosine (m^6^A) reader that binds to and stabilizes *SLC7A11* mRNA, a key regulator of cystine uptake and redox homeostasis, thereby conferring ferroptosis resistance. Importantly, pharmacological targeting of IGF2BP1 using BTYNB or Cucurbitacin B reduces tumor growth in xenograft models and decreases viability in patient‐derived gastric organoids. Together, these findings uncover a mechanism by which infection‐triggered epitranscriptomic reprogramming sustains ferroptosis resistance during gastric tumorigenesis and identify IGF2BP1 as a therapeutic target in gastric cancer.

## Graphical abstract

*Helicobacter pylori* infection induces sustained upregulation of the RNA‐binding protein IGF2BP1 in gastric epithelial cells, establishing a persistent epitranscriptomic program. IGF2BP1 stabilizes SLC7A11 mRNA in an m^6^A‐dependent manner to suppress ferroptosis and promote epithelial survival under oxidative stress. Pharmacological inhibition of IGF2BP1 reverses this ferroptosis‐resistant state and suppresses gastric tumor growth in vivo and patient‐derived organoids.

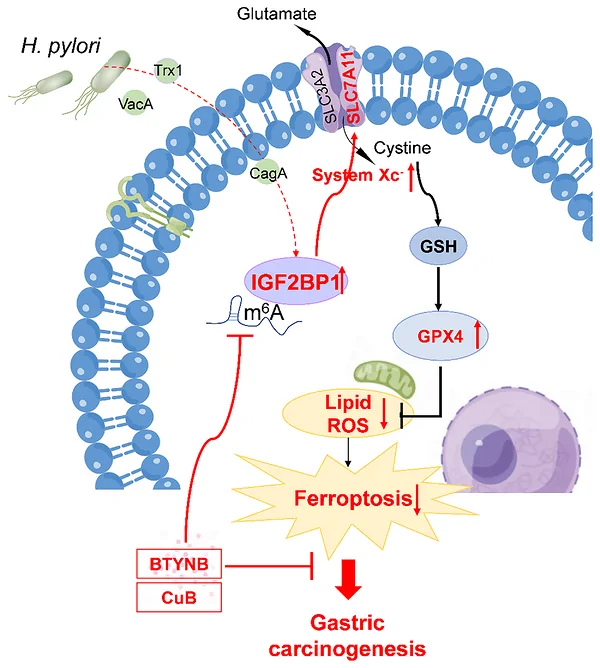

## 1 Introduction

*Helicobacter pylori* (*H. pylori*) infection is one of the most significant risk factors for the occurrence and development of gastric mucosal diseases, including gastric cancer, which remains a leading cause of cancer‐related death globally [1, 2]. Although the global prevalence of *H. pylori* infection has declined in recent decades, it is still estimated to affect approximately 43.1% of the population, and more than 90% of noncardia gastric cancer is attributed to *H. pylori* infection [3, 4]. Chronic *H. pylori* colonization is characterized by persistent inflammation and aberrant epithelial proliferation, both of which are well‐recognized drivers of gastric carcinogenesis. However, an important unresolved question is how a bacterial infection that may be transient or clinically eradicated can induce durable oncogenic reprogramming of gastric epithelial cells [5, 6]. Addressing this question is essential for understanding *H. pylori*‐associated gastric tumorigenesis and for identifying a durable therapeutic strategy.

Post‐transcriptional gene regulation has emerged as a key layer through which cells adapt to environmental and inflammatory stress. RNA‐binding proteins (RBPs) are key regulators of post‐transcriptional gene expression and play essential roles in RNA splicing, localization, stability, and translation. Dysregulation of RBP‐RNA interactions has emerged as a recurrent feature of human disease, including cancer [7]. In the progression of *H. pylori*‐induced gastric mucosal diseases, RBPs play a critical role, such as in promoting sustained bacterial infection and inhibiting host innate immune responses [8, 9]. Notably, our previous work demonstrated that *H. pylori* infection induces the expression of the RBP AU‐rich element RNA‐binding factor 1 (AUF1), leading to downregulation of gastrokine 1 (GKN1) and promotion of gastric carcinogenesis [10]. These findings raise the possibility that *H. pylori* reshapes the epithelial RBP landscape to encode long‐term pathogenic programs. However, whether infection‐induced alterations in RBPs merely reflect transient stress responses or instead encode persistent oncogenic programs remains largely unexplored.

Insulin‐like growth factor 2 mRNA‐binding protein 1 (IGF2BP1, also known as IMP1) is a highly conserved single‐stranded RBP with well‐established oncogenic functions [11]. As an RBP, IGF2BP1 serves as a post‐transcriptional fine‐tuner regulating the expression of some essential mRNA targets required for the control of tumor cell proliferation, invasion, and chemoresistance, associated with poor overall survival and metastasis in various types of human cancers [12]. Beyond tumor biology, IGF2BP1 has also been linked to host‐pathogen interactions, where it modulates viral replication and infectivity through direct RNA or protein interactions [13, 14]. More recently, IGF2BP1 has been implicated in the regulation of oxidative stress and inflammatory responses in monocytes and macrophages [15], suggesting that it may participate in broader stress‐adaptive programs activated during chronic infection. Nevertheless, whether IGF2BP1 contributes to infection‐driven oncogenic reprogramming in epithelial tissues has not been established.

Based on integrative transcriptomic and proteomic analyses, we observed that IGF2BP1 expression was markedly upregulated following *H. pylori* infection, prompting us to investigate its functional role in *H. pylori*‐associated gastric pathogenesis. Here, we demonstrated that *H. pylori* infection robustly induced IGF2BP1 expression in gastric epithelial cell lines, murine models, and human gastric tissues, and that this induction persisted even after bacterial eradication, suggesting a potential “hit‐and‐run”‐like mechanism of persistent epithelial reprogramming. Functionally, IGF2BP1 promotes epithelial survival and tumor progression by suppressing ferroptosis and enhancing oxidative stress resistance. Mechanistically, IGF2BP1 stabilizes *SLC7A11* mRNA in an m^6^A‐dependent manner, thereby maintaining redox homeostasis. Importantly, pharmacological inhibition of IGF2BP1 using BTYNB or Cucurbitacin B (CuB) suppresses tumor growth in cell models and xenograft models and reduces the viability of patient‐derived gastric cancer organoids. Collectively, our findings identify IGF2BP1 as a key mediator linking *H. pylori* infection to persistent oncogenic reprogramming and highlight its potential as a therapeutic target in *H. pylori*‐associated gastric cancer.

## 2 Results

### 2.1 *H. pylori* Infection Induces Persistent Upregulation of IGF2BP1 in Gastric Epithelium

**FIGURE 1:**
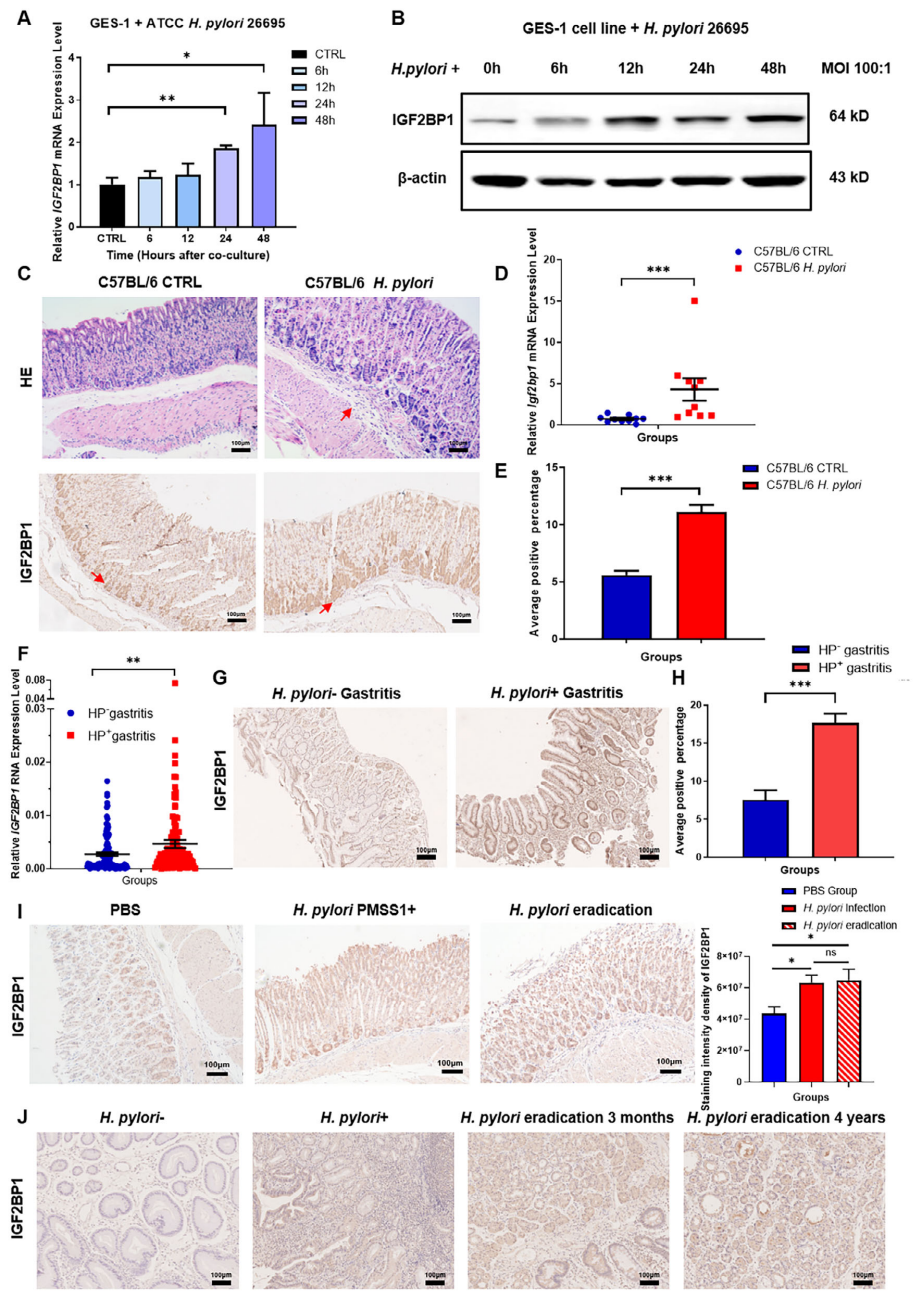
*H. pylori* infection induces persistent upregulation of IGF2BP1 in gastric epithelium. (A) The qPCR result showed a significant upregulation of IGF2BP1 following *H. pylori* 26695 infection at 6, 12, 24, and 48 h in GES‐1 cells. (B) Western blot analysis verified the time‐dependent upregulation of IGF2BP1 protein expression in infected GES‐1 cells. (C) Representative HE (up) and IGF2BP1 IHC images (down) of the *H. pylori* PMSS1‐infected group and the control group mice. Scale bars, 100 µm. (D) RT‐qPCR results showed that the mRNA level of IGF2BP1 was significantly upregulated in the *H. pylori*‐infected mice compared to the control group mice. (E) The corresponding quantitative analysis of IGF2BP1‐staining positive cells of the gastric mucosa of the *H. pylori‐*infected group and the control group mice showed a significant upregulation of IGF2BP1 protein levels in the *H. pylori*‐infected group. (F) RT‐qPCR results showed that the mRNA level of IGF2BP1 was significantly upregulated in the *H. pylori*‐infected gastritis patients compared to the *H. pylori*‐negative gastritis patients. (G) and (H) Representative IGF2BP1 IHC images of the *H. pylori*‐infected gastritis patients and *H. pylori*‐negative gastritis patients (G) and corresponding quantitative analysis of IGF2BP1‐staining positive cells of the gastric mucosa of the two groups (H). Scale bars, 100 µm. (I) IHC analysis (left) of gastric mucosa in mouse models of *H. pylori* PMSS1 infection indicated that *H. pylori* eradication did not reverse the upregulation of IGF2BP1, with both groups showing significant differences compared to the non‐infected control group (right). Scale bars, 100 µm. (J) IHC analysis of gastric biopsy samples from patients after *H. pylori* eradication showed sustained IGF2BP1 expression compared with uninfected controls, with no significant decline over time post‐eradication. All these patients’ pathological assessments were all consistent with chronic superficial gastritis. Scale bars, 100 µm. *, *p* < 0.05; **, *p* < 0.01; ***, *p* < 0.001.

We examined IGF2BP1 expression across multiple experimental systems, including gastric epithelial cell lines, chronic *H. pylori*‐infected mouse models, and gastric mucosal tissues from patients with chronic gastritis. GES‐1, BGC823, and SGC7901 cells were co‐cultured with *H. pylori* 26695 for 6, 12, 24, and 48 h. Successful infection was confirmed by immunofluorescence detection of *H. pylori* adhering to the cell membrane surface and western blot analysis of *H. pylori* protein CagA (Figure S1A,B), demonstrating efficient bacterial adhesion and consistent infection. RT‐qPCR and western blot analyses revealed a progressive increase in IGF2BP1 at both the mRNA and protein levels following *H. pylori* exposure (Figure 1A,B and Figure S2A–D), demonstrating a time‐dependent induction of IGF2BP1 in gastric epithelial cells following *H. pylori* exposure.

To validate these observations in vivo, IGF2BP1 expression was examined in a mouse model of chronic *H. pylori*‐induced gastritis. Gastric mucosal tissues were collected 12 weeks after *H. pylori* PMSS1 infection (*n* = 10 in each group). Successful *H. pylori* colonization and associated inflammatory pathology were confirmed by immunohistochemical analysis and histological evaluation (Figure S1C and Figure 1C). Both RT‐qPCR and immunohistochemical staining demonstrated a significant increase in IGF2BP1 mRNA and protein levels in *H. pylori*‐infected mice compared with uninfected controls (Figure 1C–E).

Consistent with these findings, we further collected gastric mucosal tissues from 232 patients with chronic gastritis, stratified as *H. pylori*‐negative or *H. pylori*‐positive based on rapid urease testing and Warthin‐Starry staining (*n* = 116 in each group; clinical basic characteristics are shown in Table S1). RT‐qPCR analysis revealed a significant upregulation of IGF2BP1 mRNA in *H. pylori*‐positive chronic gastritis patients (Figure 1F). Immunohistochemical staining further revealed markedly increased IGF2BP1 protein expression in *H. pylori*‐positive gastric mucosa compared with *H. pylori*‐negative controls (Figure 1G,H).

To explore whether IGF2BP1 induction represents a specific response to *H. pylori*, we treated GES‐1 cells with lipopolysaccharide (LPS), a major component of Gram‐negative bacteria. However, LPS treatment did not alter IGF2BP1 expression (Figure S2E,F), suggesting that IGF2BP1 induction is not simply a general response to bacterial endotoxin stimulation. Moreover, co‐culture with *H. pylori* 26695 strains deficient in the virulence factors CagA or VacA still resulted in significant IGF2BP1 upregulation (Figure S2G,H), indicating that IGF2BP1 induction occurs independently of these canonical virulence factors and may involve additional *H. pylori*‐specific mechanisms. To investigate the transcriptional regulation underlying IGF2BP1 induction, we performed promoter scanning using the JASPAR database. Several candidate transcription factors were identified, including AP‐1, NF‐κB‐related factors, and regulators associated with oxidative and inflammatory stress responses. Based on enrichment analysis of our transcriptomic dataset, we focused on the AP‐1 axis and found that pharmacological inhibition of AP‐1 significantly attenuated *H. pylori*‐induced IGF2BP1 upregulation (Figure S2I,J), suggesting that AP‐1‐associated signaling contributes to IGF2BP1 transcriptional activation under infection conditions.

Notably, in vivo analysis using a combined *H. pylori* infection and antibiotic eradication models demonstrated that eradication of *H. pylori* failed to reverse IGF2BP1 upregulation in the gastric mucosa (Figure 1I). Consistently, we further examined gastric mucosal tissues from patients who had undergone successful *H. pylori* eradication. The results of these patients’ pathological assessment were all consistent with chronic superficial gastritis. Immunohistochemical analysis revealed that IGF2BP1 expression remained higher than in non‐infected controls across different post‐eradication time points (Figure 1J). Given that *H. pylori*‐associated gastric carcinogenesis is thought to follow a hit‐and‐run mechanism [16], these findings indicate that *H. pylori* exposure may induce long‐lasting molecular alterations in gastric epithelium that persist beyond active infection.

### 2.2 IGF2BP1 Expression Increases Along Gastric Mucosal Disease Progression and is Associated With Poor Clinical Outcome

**FIGURE 2:**
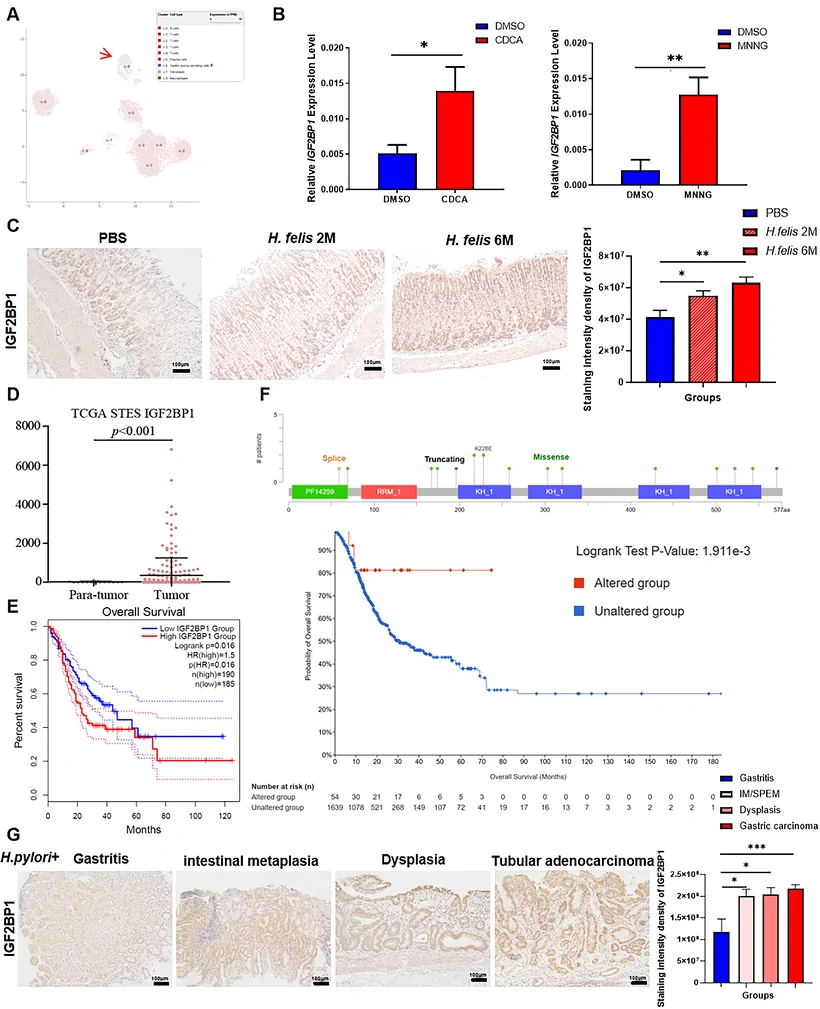
IGF2BP1 is significantly elevated with gastric mucosal disease progression and associated with poor clinical outcomes. (A) Single‐cell sequencing data from the Human Protein Atlas (HPA) database revealed that IGF2BP1 is predominantly expressed in gastric epithelial cells (cluster C‐6) within gastric tissues. (B) In GES‐1 cells with CDCA‐induced intestinal metaplasia (left) and the MNNG‐induced dysplastic model (right), the expression level of IGF2BP1 exhibited significant upregulation compared with controls. (C) Representative immunohistochemical (IHC) images (left) and quantitative analysis (right) showing increased IGF2BP1 expression in a *Helicobacter felis*‐induced mouse model of chronic gastritis and SPEM. Scale bars, 100 µm. (D) Analysis of The Cancer Genome Atlas (TCGA) dataset showing higher IGF2BP1 expression in gastric cancer tissues compared with adjacent non‐tumorous tissues. (E) Kaplan–Meier survival analysis indicating that high IGF2BP1 expression is associated with poor overall survival in gastric cancer patients. (F) cBioPortal analysis identifying IGF2BP1 mutations in gastric cancer patients, including splice‐site and truncating variants. Patients harboring IGF2BP1 alterations showed a trend toward improved clinical outcomes compared with those without detectable mutations. (G) Representative IHC images of pathological tissue samples from patients with *H. pylori*‐positive chronic superficial gastritis, intestinal metaplasia, dysplasia, and gastric cancer demonstrated a progressive increase in IGF2BP1 expression levels across disease stages. Scale bars, 100 µm. *, *p* < 0.05; **, *p* < 0.01; ***, *p* < 0.001.

Given the established role of *H. pylori* in gastric disease, we next examined the relationship between IGF2BP1 expression and gastric mucosal disease progression. Analysis of single‐cell sequencing data from the Human Protein Atlas (HPA) database (https://www.proteinatlas.org/) revealed that IGF2BP1 is predominantly expressed in gastric epithelial cells within gastric tissues (Figure 2A), suggesting a potential epithelial‐specific function.

We next assessed IGF2BP1 expression in experimental models representing different stages of gastric precancerous lesions. In chenodeoxycholic acid (CDCA)‐induced intestinal metaplasia models and N‐methyl‐N′‐nitro‐N‐nitrosoguanidine (MNNG)‐induced dysplastic GES‐1 cells, IGF2BP1 expression was significantly increased compared with controls (Figure 2B). To further validate these findings in vivo, a *Helicobacter felis* (*H. felis*)‐induced mouse model of chronic gastritis and spasmolytic polypeptide‐expressing metaplasia (SPEM) was established [17, 18, 19]. Histological analysis by HE staining and immunofluorescence confirmed progressive pathological changes. At 2 months post‐infection, gastric lesions are primarily characterized by chronic inflammation with massive infiltration of inflammatory cells, whereas at 6 months, more advanced pathological features consistent with SPEM are observed (Figure S3). Consistently, immunohistochemical staining revealed a stepwise increase in IGF2BP1 expression during disease progression (Figure 2C).

We next evaluated IGF2BP1 expression in human gastric cancer cohorts. Analysis of The Cancer Genome Atlas (TCGA) dataset demonstrated significantly higher IGF2BP1 expression in gastric cancer tissues compared with matched adjacent non‐tumorous tissues (Figure 2D). Kaplan–Meier survival analysis further revealed that high IGF2BP1 expression was significantly associated with poorer overall survival in patients with gastric cancer (Figure 2E). In addition, interrogation of the cBioPortal database identified IGF2BP1 mutations, including splice‐site and truncating variants, in a subset of gastric cancer patients, providing genomic context supporting the clinical relevance of IGF2BP1. Notably, patients harboring mutated IGF2BP1 exhibited improved clinical outcomes compared with those with wild‐type IGF2BP1 (Figure 2F), indicating that disruption of IGF2BP1 function may be associated with reduced tumor aggressiveness.

To further validate the association between IGF2BP1 expression and gastric disease progression, we performed immunohistochemical staining of gastric tissue specimens from patients with *H. pylori*‐positive chronic superficial gastritis, intestinal metaplasia, dysplasia, and gastric cancer. IGF2BP1 expression increased progressively across these pathological stages, paralleling disease severity (Figure 2G). Collectively, these data demonstrate that elevated IGF2BP1 expression accompanies gastric mucosal disease progression and is associated with adverse clinical outcomes.

### 2.3 IGF2BP1 Promotes Epithelial Proliferation and Tumorigenic Phenotypes

**FIGURE 3:**
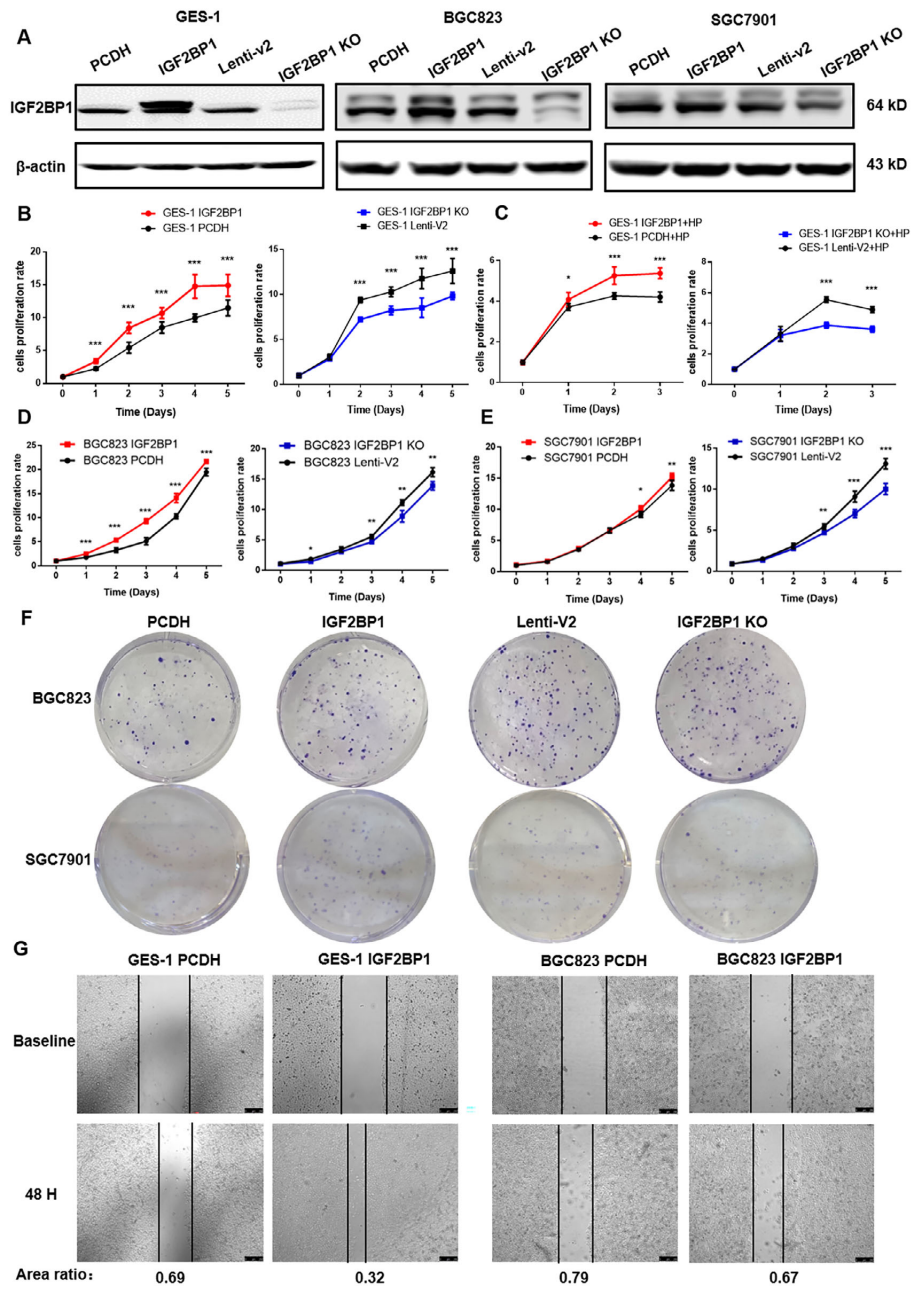
Elevated IGF2BP1 promotes cell proliferation and tumor‐associated phenotypes in gastric epithelial and cancer cells. (A) Western blot analysis verified that stable cell lines with either IGF2BP1 overexpression or knockout were generated in GES‐1, BGC823, and SGC7901 cells. The PCDH group was infected with an overexpressing blank control plasmid and therefore served as the control for the IGF2BP1 overexpression group, whereas the Lenti‐V2 group was infected with a Lenti‐CRISPR/Cas9 empty vector and served as the control for the IGF2BP1 knockout group. (B) CCK‐8 assays revealed that IGF2BP1 overexpression enhanced cell proliferation (left), while IGF2BP1 knockout suppressed it in GES‐1 cells (right). (C) Under *H. pylori* co‐culture conditions, IGF2BP1‐overexpressing GES‐1 cells maintained enhanced proliferative capacity, while IGF2BP1‐deficient cells exhibited reduced growth. (D) and (E) The same results were confirmed in the BGC823 (D) and SGC7901 (E) cell lines, which indicated that IGF2BP1 also promoted the proliferation of cancer cells. (F) Colony formation assays revealed that overexpression of IGF2BP1 significantly enhanced tumor cell proliferation. (G) Scratch assays demonstrated that IGF2BP1 overexpression notably increased tumor cell migration. *, *p* < 0.05; **, *p* < 0.01; ***, *p* < 0.001.

To investigate the functional consequences of IGF2BP1 upregulation, we generated stable gastric epithelial and gastric cancer cell lines with stable IGF2BP1 overexpression or CRISPR/Cas9‐mediated knockout. To characterize the basal expression landscape of IGF2BP1, we first performed western blot analysis across commonly used gastric epithelial and cancer cell lines (Figure S4A), which supported subsequent model selection. GES‐1 cells were used to model early epithelial responses to *H. pylori* infection, whereas BGC823 and SGC7901 cells were used to determine whether IGF2BP1‐dependent regulatory mechanisms are preserved in malignant contexts. Lentiviral pCDH‐IGF2BP1‐3×Flag and Lenti‐CRISPR/Cas9‐IGF2BP1 constructs were introduced into GES‐1, BGC823, and SGC7901 cells, and successful modulation of IGF2BP1 expression was confirmed by western blot analysis (Figure 3A).

We first assessed cell proliferation in GES‐1 cells using CCK‐8 assays. IGF2BP1 overexpression significantly increased cell proliferation, whereas IGF2BP1 depletion markedly suppressed proliferative capacity (Figure 3B). Notably, under *H. pylori* co‐culture conditions, IGF2BP1‐overexpressing cells maintained a pronounced proliferative advantage compared with control cells, while IGF2BP1‐deficient cells exhibited reduced growth (Figure 3C). These results indicate that IGF2BP1 promotes epithelial cell survival and proliferation under infection‐associated stress conditions. Consistent results were observed in the gastric cancer cell lines BGC823 and SGC7901, in which IGF2BP1 overexpression similarly enhanced cell proliferation, while IGF2BP1 knockout exerted an inhibitory effect (Figure 3D,E), indicating a conserved pro‐tumorigenic role of IGF2BP1 across gastric epithelial and cancer contexts.

We further evaluated whether IGF2BP1 influences additional tumor‐associated cellular phenotypes. Colony formation assays demonstrated that IGF2BP1 overexpression significantly increased clonogenic growth capacity (Figure 3F). In addition, wound‐healing assays revealed that IGF2BP1 overexpression enhanced the migratory potential of gastric cancer cells compared with control cells (Figure 3G). Collectively, these findings demonstrate that IGF2BP1 promotes proliferative, survival, and migratory phenotypes in gastric epithelial and cancer cells, and enhances cellular fitness under *H. pylori* infection‐associated stress.

### 2.4 IGF2BP1 Limits Oxidative Stress and Protects Gastric Epithelial Cells From Ferroptosis

Oxidative stress triggered by reactive oxygen species (ROS) plays a critical role in host‐pathogen interactions during *H. pylori* infection. Although *H. pylori*‐associated oxidative stress has been extensively studied, the mechanisms by which gastric epithelial cells regulate redox homeostasis and cell death pathways in response to infection remain incompletely understood [20, 21]. Emerging evidence suggests that perturbations in redox balance induced by *H. pylori* may predispose gastric epithelial cells to ferroptosis [22]; however, it remains unclear whether IGF2BP1 participates in this process.

**FIGURE 4:**
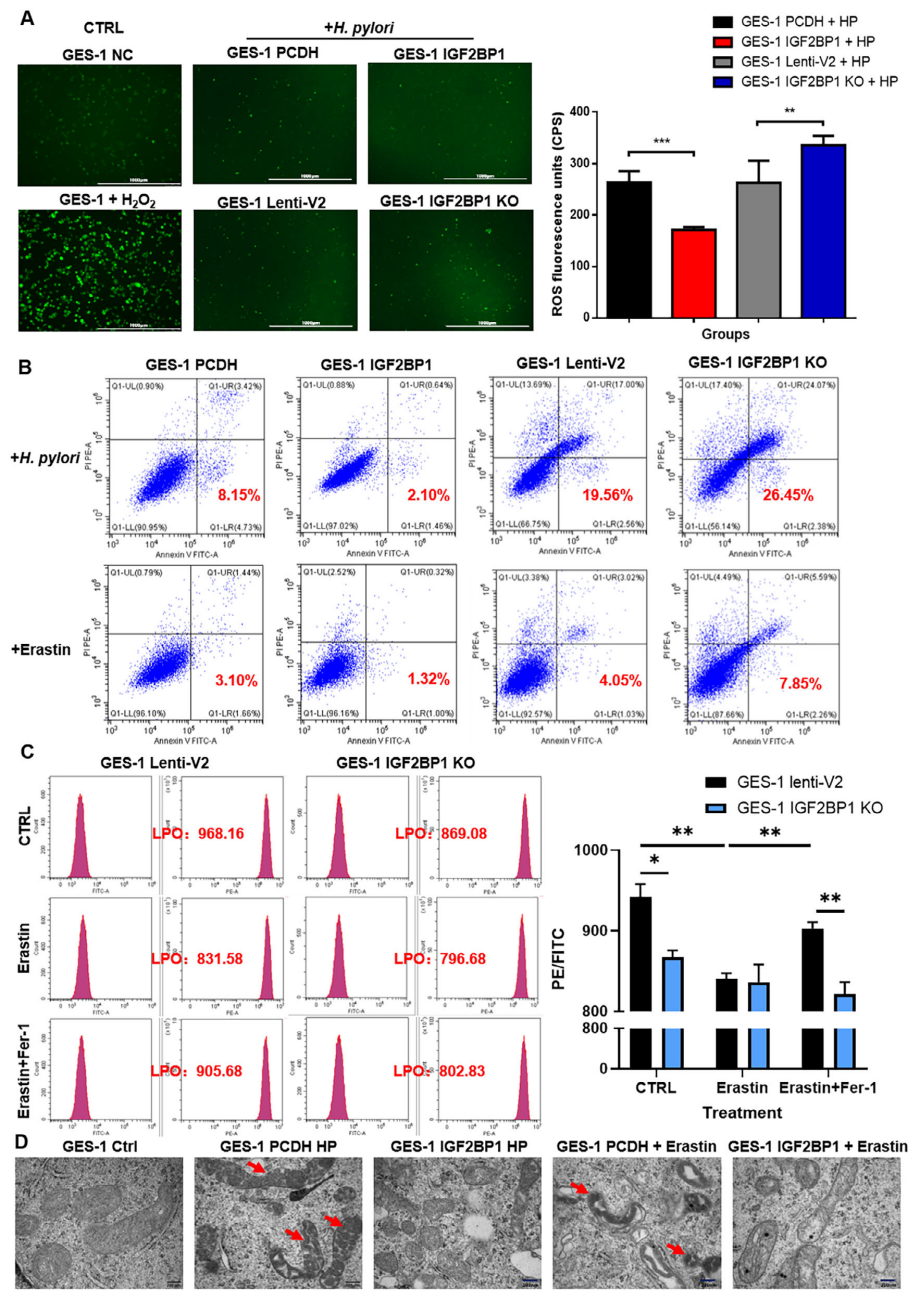
IGF2BP1 attenuates ROS accumulation and suppresses ferroptosis in gastric epithelial cells. (A) Representative fluorescence images (left) and quantitative analysis (right) of ROS levels in IGF2BP1‐overexpressing and knockout cell lines co‐cultured with *H. pylori* using the DCFH‐DA probe. IGF2BP1 overexpression significantly reduced ROS levels, while IGF2BP1 knockout led to a marked increase in ROS production. GES‐1cells treated with H_2_O_2_ were used as the positive control in the experiment. Scale bars, 1000 µm. (B) Flow cytometry results showed that overexpression of IGF2BP1 significantly inhibited cell death induced by *H. pylori* and erastin, and in contrast, IGF2BP1 knockdown significantly enhanced cell sensitivity. (C) Flow cytometry analysis of lipid peroxidation showed that knockout of IGF2BP1 enhanced erastin‐induced lipid peroxidation in cells. Fer‐1 effectively rescued erastin‐induced ferroptosis in the Lenti‐V2 control cells. Following IGF2BP1 knockout, cells exhibited enhanced sensitivity to ferroptosis; although Fer‐1 still conferred partial protection, the overall survival capacity remained significantly reduced. (D) Representative images of transmission electron microscopy revealed a clear ferroptosis phenotype in the control cells upon addition of *H. pylori* or erastin, characterized by significant mitochondrial swelling, increased membrane density, and the disappearance of mitochondrial cristae, while overexpression of IGF2BP1 notably alleviated this phenomenon. Scale bars, 200 nm. **, *p* < 0.01; ***, *p* < 0.001.

To assess whether IGF2BP1 regulates oxidative stress, intracellular ROS levels were measured in IGF2BP1‐overexpressing and IGF2BP1‐knockout cells co‐cultured with *H. pylori* using the DCFH‐DA fluorescent probe. IGF2BP1 overexpression significantly reduced ROS accumulation, whereas IGF2BP1 knockout resulted in a marked increase in ROS production (Figure 4A), indicating that IGF2BP1 contributes to the maintenance of redox homeostasis under infection‐associated stress conditions.

Given the established link between oxidative stress and ferroptosis, we next evaluated whether IGF2BP1 modulates ferroptosis. Cells with altered IGF2BP1 expression were exposed to *H. pylori* or the ferroptosis inducer erastin. IGF2BP1 overexpression significantly attenuated erastin‐ and *H. pylori*‐induced cell death, whereas IGF2BP1 depletion markedly sensitized cells to these stimuli (Figure 4B and Figure S5A). Consistent with this observation, lipid peroxidation, a hallmark of ferroptosis, was significantly increased in IGF2BP1‐deficient cells following erastin treatment, as assessed by lipid peroxidation (LPO) assays (Figure 4C and Figure S6A–C). To further confirm that the observed phenotype is ferroptosis‐dependent, rescue experiments were performed using the ferroptosis inhibitor ferrostatin‐1 (Fer‐1). In control cells, Fer‐1 effectively rescued erastin‐induced ferroptosis (Figure 4C and Figure S6A–C). However, following IGF2BP1 knockout, cells exhibited enhanced sensitivity to ferroptosis; although Fer‐1 still conferred partial protection, the overall survival capacity remained significantly reduced (Figure 4C and Figure S6A–C), indicating that IGF2BP1 is a key regulator of ferroptosis susceptibility under oxidative stress conditions.

To further substantiate the involvement of ferroptosis at the ultrastructural level, transmission electron microscopy revealed characteristic ferroptosis changes in control cells exposed to *H. pylori* or erastin, including pronounced mitochondrial swelling, increased membrane density, and loss of mitochondrial cristae. In contrast, these mitochondrial abnormalities were markedly alleviated in IGF2BP1‐overexpressing cells, which displayed ultrastructural features comparable to untreated controls (Figure 4D).

Together, these data demonstrate that IGF2BP1 limits oxidative stress and suppresses ferroptosis in gastric epithelial cells, thereby promoting cell survival under *H. pylori*‐associated oxidative stress conditions.

### 2.5 IGF2BP1 Confers Ferroptosis Resistance by Stabilizing *SLC7A11* mRNA in an *N^6^*‐Methyladenosine (m^6^A)‐Dependent Manner

To identify downstream effectors mediating the impact of IGF2BP1 on oxidative stress and ferroptosis, transcriptome profiling was performed in stable IGF2BP1‐overexpressing cells and compared with vector controls (Figure S7A,B). These data were integrated with our previously generated RNA‐sequencing dataset from *H. pylori*‐infected gastric epithelial cells (Figure S7C,D). Intersection analysis identified 245 overlapping differentially expressed genes (DEGs; fold change ≥2) shared between IGF2BP1 overexpression and *H. pylori* infection (Figure S8A).

**FIGURE 5:**
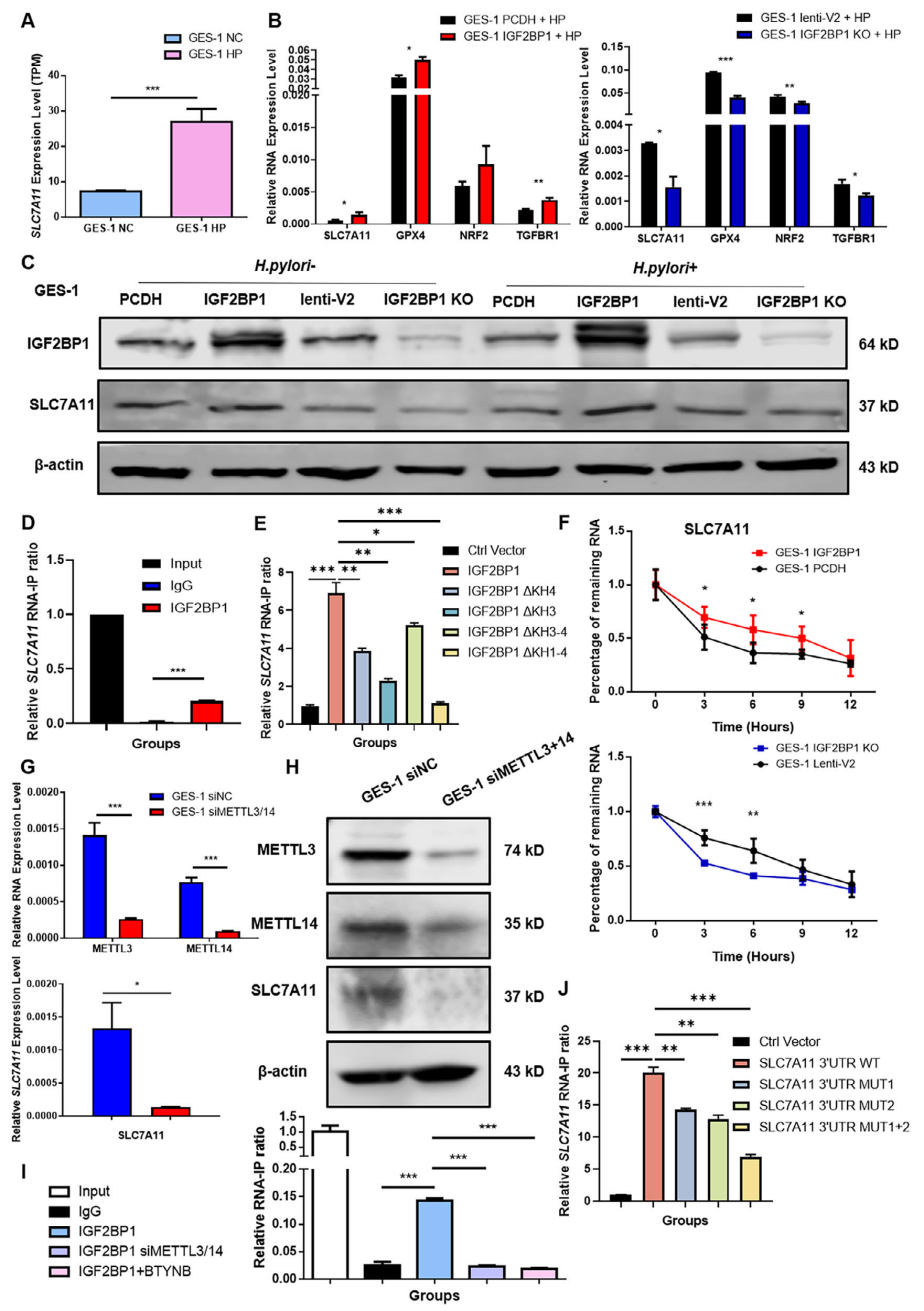
IGF2BP1 promotes SLC7A11 expression by stabilizing its mRNA in an m^6^A‐dependent manner. (A) Transcriptomic analysis revealed that SLC7A11 was significantly upregulated. (B) RT‐qPCR results showed that the level of *SLC7A11*, *NFE2L2*, *GPX4*, and *TGFBR1* was significantly upregulated when IGF2BP1 was overexpressed and decreased when IGF2BP1 was knocked down. (C) Western blot results showed that SLC7A11 protein levels were significantly upregulated after IGF2BP1 overexpression and downregulated when IGF2BP1 was knocked out. (D) RNA immunoprecipitation (RIP) assays confirmed that IGF2BP1 could bind to the *SLC7A11* mRNA. (E) RIP assays using IGF2BP1 truncation mutants revealed that deletion of the KH domain markedly abolishes IGF2BP1 binding to *SLC7A11* mRNA, indicating that the RNA‐binding domain is essential for this interaction. (F) RNA turnover experiments verified that IGF2BP1 enhanced *SLC7A11* mRNA stability. (G) The RT‐qPCR results showed a significant decrease in *SLC7A11*, *METTL3*, and *METTL14* after knocking down the methyltransferases METTL3 and METTL14. (H) Western blot analysis verified the significant decrease in SLC7A11, METTL3, and METTL14 proteins after knocking down the methyltransferases METTL3 and METTL14. (I) RIP assays showing that inhibition of m^6^A modification, including METTL3/14 knockdown or BTYNB treatment, significantly reduces IGF2BP1 binding to SLC7A11 mRNA. (J) RIP assays using SLC7A11 mRNA mutants with disrupted m6A consensus sites demonstrated markedly reduced IGF2BP1 binding, confirming that the IGF2BP1‐SLC7A11 interaction is m^6^A‐dependent. *, *p* < 0.05; **, *p* < 0.01; ***, *p* < 0.001.

Gene Ontology enrichment analysis and KEGG pathway analysis revealed that these DEGs were predominantly associated with membrane transport processes and inflammatory signaling pathways (Figure S8B,C). Although multiple oxidative stress‐related genes were identified in these candidates (Figure S7A), SLC7A11 uniquely represents the central rate‐limiting component of the cystine‐glutamate antiporter system, which directly determines intracellular glutathione biosynthesis and ferroptosis sensitivity (Figure 5A). In addition, CHAC1, another key regulator of glutathione metabolism, further supports the enrichment of ferroptosis‐associated programs within the intersection set.

SLC7A11 encodes the light‐chain subunit of the cystine/glutamate antiporter system Xc^−^, which mediates cystine uptake and plays a central role in maintaining cellular redox homeostasis and preventing ferroptosis [23, 24]. RT‐qPCR analysis demonstrated that *SLC7A11* mRNA levels were significantly increased upon IGF2BP1 overexpression and markedly reduced following IGF2BP1 depletion (Figure 5B). Consistently, SLC7A11 protein expression exhibited parallel changes in response to IGF2BP1 modulation (Figure 5C). In addition, the expression levels of key components associated with the SLC7A11 axis, including *NFE2L2* (NRF2), *GPX4*, and *TGFBR1*, were also modulated in response to IGF2BP1 alteration (Figure 5B), supporting a broader functional link between IGF2BP1 and ferroptosis‐associated signaling. Upon IGF2BP1 knockout, the SLC7A11 level was significantly downregulated, which further enhanced erastin‐induced suppression of SLC7A11 and its downstream effector GPX4. Treatment with ferrostatin‐1 (Fer‐1) partially restored SLC7A11 expression and attenuated its downregulation; however, levels remained lower than those in control cells (Figure S9). Collectively, these findings indicated the IGF2BP1‐SLC7A11 regulatory axis as a central epitranscriptomic pathway that preserves redox homeostasis and suppresses ferroptosis in gastric epithelial cells under *H. pylori*‐associated stress.

Given that IGF2BP1 functions as an m^6^A reader protein regulating mRNA stability [25, 26, 27], we next examined whether SLC7A11 is directly regulated by IGF2BP1 in an m^6^A‐dependent manner. Bioinformatic analysis using the SRAMP database (http://www.cuilab.cn/sramp) identified ten putative m^6^A modification sites within the *SLC7A11* transcript, two of which overlapped with reported IGF2BP1 recognition motifs (Figure S10A). RNA immunoprecipitation (RIP) assays confirmed a direct association between IGF2BP1 and *SLC7A11* mRNA (Figure 5D and Figure S10B). Besides, given that the RNA‐binding capacity of IGF2BP1 is primarily mediated by its KH (K‐homology) domains [28, 29], we generated IGF2BP1 constructs harboring KH‐domain‐deficient mutations and performed RIP assays to assess their ability to bind *SLC7A11* mRNA (Figure S10C). Our results demonstrated that wild‐type IGF2BP1 robustly bound *SLC7A11* transcripts, whereas mutation of the KH domain leads to a marked reduction in IGF2BP1‐SLC7A11 binding (Figure 5E and Figure S10D), indicating that the KH domain is essential for target recognition. This result further confirms that the truncated form of IGF2BP1 exhibits reduced function, with patients showing a more favourable survival curve. Moreover, RNA stability assays following transcriptional blockade with actinomycin D revealed that *SLC7A11* mRNA decay was significantly delayed in IGF2BP1‐overexpressing cells, whereas IGF2BP1 depletion markedly accelerated *SLC7A11* mRNA degradation (Figure 5F), indicating that IGF2BP1 enhances *SLC7A11* mRNA stability.

To determine whether this interaction depends on m^6^A methylation, the core m^6^A methyltransferases METTL3 and METTL14 were silenced (Figure 5G,H), resulting in a global reduction in m^6^A modification levels (Figure S10E). Under these conditions, both *SLC7A11* mRNA and protein expression were significantly decreased (Figure 5G,H). Notably, RIP assays demonstrated a substantial reduction in IGF2BP1 binding to *SLC7A11* mRNA upon METTL3/14 knockdown (Figure 5I and Figure S10F). To further validate the essentiality of site‐specific m^6^A modification, we generated vectors containing wild‐type, single‐ or double‐point mutant predicted m^6^A consensus sites within the SLC7A11 3’UTR. RIP assays were then performed following transfection of these vectors, and results demonstrated that mutation of the predicted m^6^A sites led to a marked and reproducible reduction in IGF2BP1 binding to *SLC7A11* mRNA (Figure 5J and Figure S10G), indicating that IGF2BP1 recognizes SLC7A11 in an m^6^A‐dependent manner. These findings together provided complementary evidence that m^6^A modification is required for efficient IGF2BP1 binding to SLC7A11 transcripts.

Finally, to evaluate the functional requirement of SLC7A11 in gastric epithelial cells, we attempted to generate stable SLC7A11 knockout cell lines using the CRISPR/Cas9 system. Although efficient SLC7A11 disruption was confirmed (Figure S11A), we were unable to obtain viable stable knockout clones despite extensive screening and supplementation with the reducing agent β‐mercaptoethanol. These observations are consistent with an essential role of SLC7A11 in maintaining cellular redox balance and survival in gastric epithelial and gastric cancer cells. Therefore, we then performed transient siRNA‐mediated knockdown of SLC7A11, which efficiently reduced both mRNA and protein expression (Figure S11B,C). Importantly, we further evaluated whether SLC7A11 knockdown could reverse the functional effects induced by IGF2BP1 overexpression. Our results demonstrated that IGF2BP1 overexpression suppresses ferroptosis‐related phenotypes under *H. pylori* stimulation by promoting the expression of molecules downstream of the SLC7A11 pathway, whereas silencing of SLC7A11 attenuates these IGF2BP1‐mediated protective effects (Figure S11D). These findings indicate that the pro‐survival and anti‐ferroptosis effects of IGF2BP1 are at least partially dependent on SLC7A11 activity.

### 2.6 Pharmacological Inhibition of IGF2BP1 Suppresses Gastric Tumor Growth In Vivo and in Patient‐Derived Organoids

**FIGURE 6:**
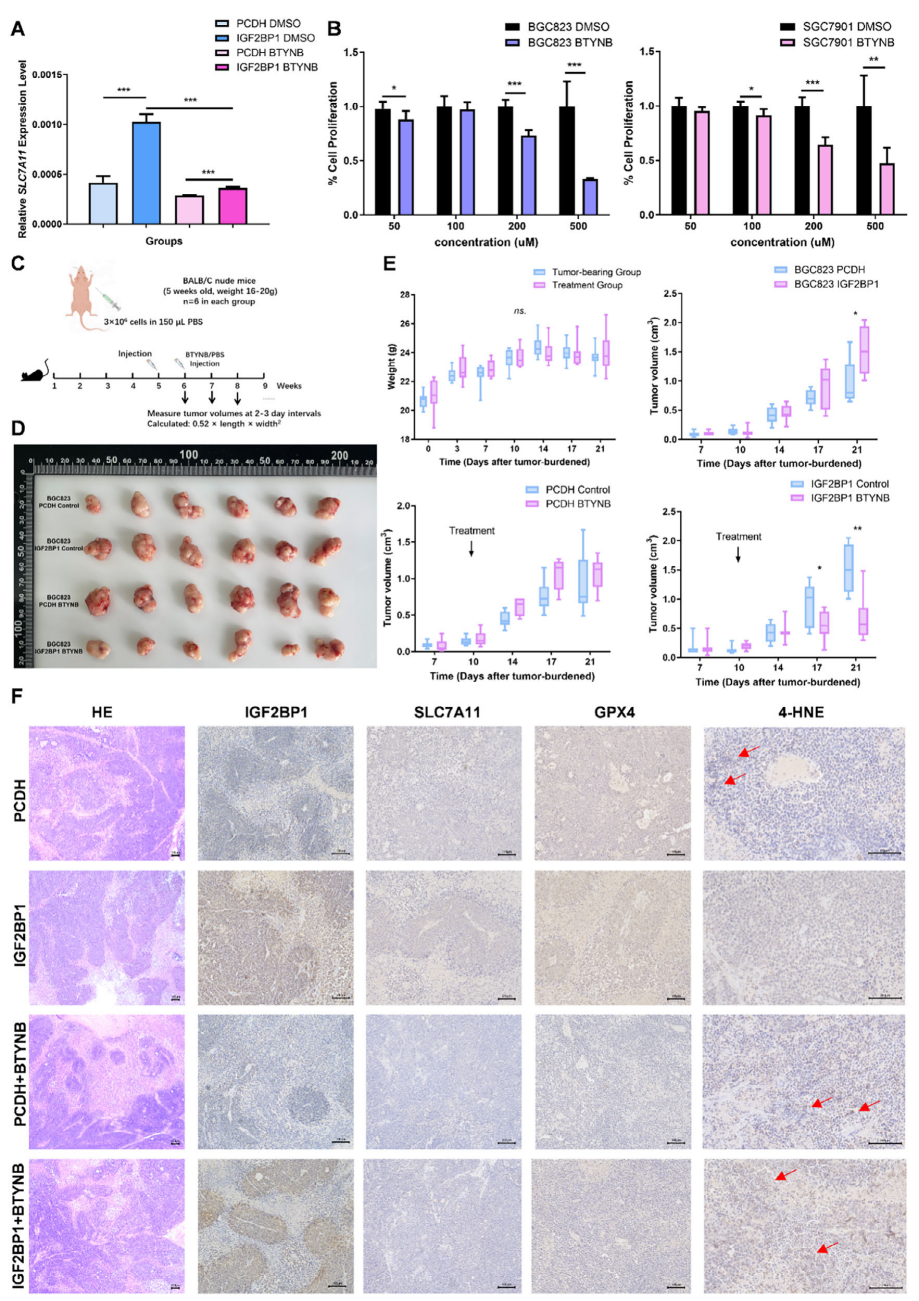
The small molecule BTYNB significantly inhibited the proliferation of gastric cells and the xenograft model. (A) RT‐qPCR results revealed that BTYNB significantly inhibited the expression level of *SLC7A11* even with the overexpression of IGF2BP1. (B) CCK‐8 assay showed that BTYNB significantly inhibited tumor cell proliferation in the BGC823 and SGC7901 cells in a dose‐dependent manner. (C) The mouse experimental scheme. BALB/C nude mice were subcutaneously injected with the gastric cancer cell line BGC823 (3 × 10^6^ cells per tumor) and followed by PBS or BTYNB intratumoral injection, respectively, one week after tumor formation. The tumor size and body weight were measured at 2‐or 3‐day intervals. Four groups of mice (*n* = 6 in each group) were sacrificed 3 weeks later. (D) The tumor tissue from each group of mice. (E) There was no significant difference in the body weights of the mice in each group. Overexpression of IGF2BP1 resulted in enhanced tumor growth, while intratumoral injection of BTYNB significantly suppressed tumor cell growth, particularly in the IGF2BP1‐overexpressing groups. (F) Representative HE and IHC images of IGF2BP1, SLC7A11, GPX4, and 4‐HNE staining of the gastric tumor of the four‐group mice. The results confirmed that the levels of SLC7A11 and GPX4 were significantly upregulated in the tumor tissues of the IGF2BP1 overexpression group. Following BTYNB treatment, intratumoral 4‐HNE accumulation increased, suggesting that IGF2BP1 inhibition promotes ferroptosis in tumor cells, resulting in improved therapeutic efficacy. Scale bars, 100 µm and 200 µm. *, *p* < 0.05; **, *p* < 0.01; ***, *p* < 0.001.

2‐{[(5‐bromo‐2‐thienyl) methylene] amino} benzamide (BTYNB) is a structure‐specific small‐molecule inhibitor that disrupts the RNA‐binding activity of IGF2BP1 by interfering with its interaction with target transcripts [30, 31]. To evaluate the therapeutic relevance of IGF2BP1 in *H. pylori*‐associated gastric tumorigenesis, BTYNB was applied to stable IGF2BP1‐overexpressing gastric epithelial and cancer cell models. BTYNB treatment resulted in a marked reduction in the *SLC7A11* mRNA level (Figure 6A). Consistently, RIP assays demonstrated a significant decrease in the association between IGF2BP1 and *SLC7A11* mRNA following BTYNB treatment (Figure 5I and Figure S10F), confirming that BTYNB interferes with IGF2BP1‐RNA interactions at the post‐transcriptional level.

We next assessed the impact of IGF2BP1 inhibition on gastric cancer cell growth. In BGC823 and SGC7901 cells, BTYNB treatment significantly suppressed cell growth in a dose‐dependent manner (Figure 6B). To further evaluate the anti‐tumor efficacy of BTYNB in vivo, a nude mouse xenograft model was established using gastric cancer cells with enforced IGF2BP1 expression (Figure 6C). As expected, IGF2BP1 overexpression markedly accelerated tumor growth. Notably, intratumoral administration of BTYNB significantly inhibited tumor progression, with the most pronounced inhibitory effect observed in tumors exhibiting high IGF2BP1 expression (Figure 6D,E). Mechanistically, immunohistochemical analysis of xenograft tissues revealed elevated expression of SLC7A11 and GPX4 in IGF2BP1‐overexpressing tumors, consistent with suppressed ferroptotic activity (Figure 6F). In contrast, BTYNB‐treated tumors displayed a marked increase in intratumoral 4‐hydroxynonenal (4‐HNE) accumulation, a lipid peroxidation marker indicative of ferroptosis activation (Figure 6F). These findings indicate that BTYNB selectively impairs IGF2BP1‐dependent ferroptosis resistance, thereby restraining tumor growth in vivo.

**FIGURE 7:**
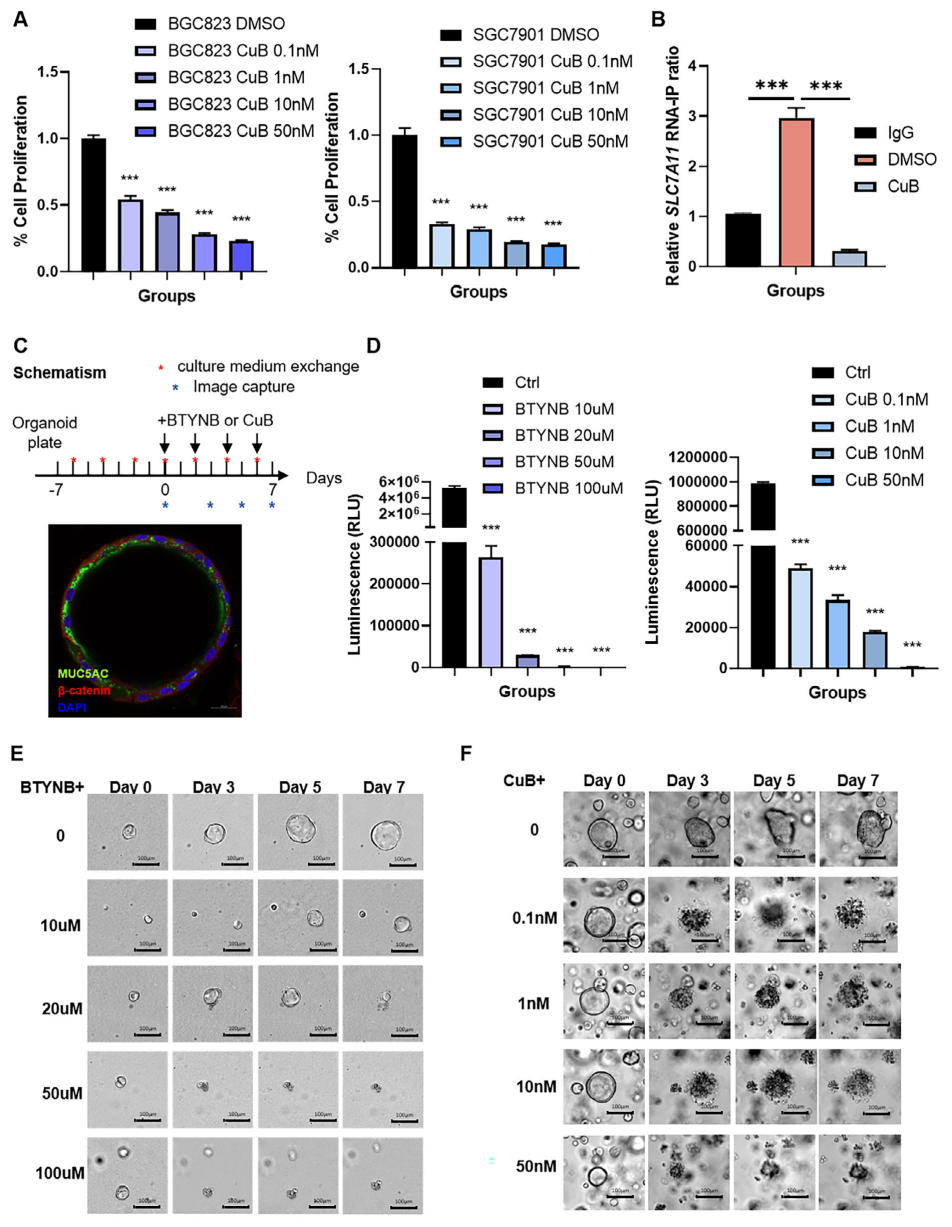
The BTYNB and CuB suppress gastric tumor growth in the patient‐derived organoids. (A) Dose‐dependent inhibitory effects of cucurbitacin B (CuB) on cell viability in gastric cancer cell lines BGC823 and SGC7901. (B) RNA immunoprecipitation (RIP) assays demonstrating that CuB significantly reduces the binding between IGF2BP1 and *SLC7A11* mRNA. (C) Schematic presentation of experimental procedures for the patient‐derived organoids treated with BTYNB or CuB. (D) Results from 3D cell viability assays show that both BTYNB (left) and CuB (right) significantly inhibit patient‐derived organoid (*n* = 3, with three independent biological replicates for each sample) survival in a dose‐dependent manner. (E) and (F) High‐content imaging results suggested that the addition of BTYNB (E) or CuB (F) led to a significant inhibition of gastric cancer patient‐derived organoid (*n* = 3, with three independent biological replicates for each sample) growth, reduced organoid size, and induced fragmentation and cell death in a time‐ and dose‐dependent manner. Scale bars, 100 µm. ***, *p* < 0.001.

Given the limited information regarding the clinical safety profile and used at the high dose of BTYNB, we further explored the therapeutic relevance of targeting IGF2BP1 using another independent pharmacological approach, cucurbitacin B (CuB), a bioactive component of the traditional Chinese medicine formulation Cucurbitacin Tablets, which has been reported that could modulate IGF2BP1 activity in hepatitis and hepatocellular carcinoma [32, 33]. In BGC823 and SGC7901 cells, CuB treatment significantly suppressed cell growth in a dose‐dependent manner (Figure 7A). Furthermore, it exhibited a significant inhibitory effect on cell proliferation in the IGF2BP1 overexpression group in a manner comparable to the control group (Figure S12A). Importantly, RIP assays further demonstrated that CuB treatment significantly reduced the interaction between IGF2BP1 and *SLC7A11* mRNA (Figure 7B), suggesting that CuB could disrupt IGF2BP1‐mediated RNA‐binding activity.

Finally, to assess clinical translational relevance, patient‐derived gastric cancer organoids were established to evaluate drug responses in a clinically relevant ex vivo system (*n* = 3, Figure 7C). Both BTYNB and CuB significantly reduced organoid viability, as assessed by CellTiter‐Glo 3D assays (Figure 7D), and induced pronounced morphological alterations characterized by reduced organoid size, structural fragmentation, and increased cell death, as revealed by high‐content imaging analysis (Figure 7E,F). These effects were observed in a time‐ and dose‐dependent manner.

Collectively, these results demonstrate that pharmacological inhibition of IGF2BP1 using two independent small‐molecule strategies disrupts IGF2BP1‐RNA interactions, suppresses SLC7A11‐mediated ferroptosis resistance, and effectively suppresses gastric cancer growth in vitro, in vivo, and in patient‐derived organoids. These findings identify IGF2BP1 as a therapeutically actionable target in gastric cancers characterized by elevated IGF2BP1 expression.

## 3 Discussion

Although widespread eradication strategies have reduced infection rates of *H. pylori*, the increasing prevalence of antibiotic‐resistant *H. pylori* strains and the incidence of gastric cancer after *H. pylori* eradication highlight the limitations of eradication‐based approaches and underscore the need to define host‐intrinsic mechanisms that sustain cancer risk following infection [34, 35]. In this study, we identify RNA‐binding protein IGF2BP1 as a central host factor linking *H. pylori* infection to sustained gastric tumorigenesis. We show that *H. pylori* infection robustly induces IGF2BP1 expression across in vitro systems, murine models, and human gastric tissues. Notably, elevated IGF2BP1 expression persists even after bacterial eradication, in both mouse models and patient samples, supporting a “hit‐and‐run” paradigm whereby transient microbial exposure results in durable oncogenic reprogramming of the gastric epithelium [16]. This persistent upregulation of IGF2BP1 provides a potential mechanistic explanation for the continued elevation of gastric cancer risk following successful *H. pylori* clearance.

Functionally, our data indicate that IGF2BP1 promotes gastric epithelial cell survival and disease progression primarily by attenuating oxidative stress and suppressing ferroptosis. Ferroptosis is increasingly recognized as a tumor‐suppressive form of regulated cell death, particularly in epithelial tissues, yet its regulation in the context of chronic bacterial infection has remained poorly defined. Our findings suggest that *H. pylori* infection engages an RNA‐binding protein‐mediated program that enables gastric epithelial cells to evade ferroptosis, thereby facilitating long‐term survival under oxidative stress conditions associated with chronic inflammation.

At the mechanistic level, we demonstrate that IGF2BP1 functions as an m^6^A reader to bind and stabilize *SLC7A11* mRNA in an m^6^A‐dependent manner. SLC7A11 encodes the light chain of the cystine/glutamate antiporter system Xc^−^, a key regulator of intracellular redox homeostasis and ferroptosis sensitivity. Single‐cell RNA‐seq data from the HPA database indicate that SLC7A11 is selectively enriched in gastric epithelial cells compared with other cell types, suggesting that it is a key component of epithelial redox regulation. Notably, although its absolute expression level is not exceptionally high, SLC7A11 functions as a rate‐limiting determinant of cystine uptake and redox homeostasis, implying that even modest changes in its expression may exert disproportionate effects on cellular oxidative stress balance and ferroptosis sensitivity. By enhancing *SLC7A11* mRNA stability, IGF2BP1 increases cystine uptake, limits lipid peroxidation, and suppresses ferroptosis, thereby promoting cell proliferation and tumor progression in *H. pylori*‐associated gastric disease. These findings place the IGF2BP1‐SLC7A11 axis at the center of an infection‐driven epitranscriptomic program that enables gastric epithelial cells to evade ferroptosis.

Although we sought to define upstream bacterial determinants responsible for IGF2BP1 induction, deletion of the canonical *H. pylori* virulence factors, including CagA and VacA, did not abolish IGF2BP1 upregulation. Our further results suggested that IGF2BP1 is likely regulated by a broader inflammatory transcriptional network. In particular, AP‐1‐associated signaling downstream of TLR and MAPK activation emerges as a major contributor to IGF2BP1 upregulation, consistent with a model in which infection‐induced inflammatory and oxidative stress signals converge on AP‐1 to drive persistent IGF2BP1 expression. Future studies are required to further dissect the relative contributions of NF‐κB, AP‐1, and oxidative stress pathways in establishing long‐term IGF2BP1 activation.

IGF2BP1 is a highly conserved RNA‐binding protein with well‐established roles in post‐transcriptional regulation, including mRNA transport, stability, and translation [36, 37]. As an oncofetal protein, IGF2BP1 is minimally expressed in most adult tissues but frequently reactivated in cancer [11]. Since its identification as an m^6^A reader, IGF2BP1 has been shown to regulate a broad repertoire of m^6^A‐modified transcripts involved in cell growth, migration, and therapy resistance [26, 38, 39]. While previous studies have implicated IGF2BP1 in gastric cancer through interactions with long non‐coding RNAs [40, 41, 42], we acknowledge additional factors in the tumor microenvironment may also contribute to IGF2BP1 regulation in gastric cancer, our findings clarify that *H. pylori* infection is a major but not exclusive driver of IGF2BP1 upregulation and extend its functional scope by implicating IGF2BP1 as a key regulator of ferroptosis in the setting of infection‐associated gastric tumorigenesis. Recently, Chen et al. reported that *H. pylori* infection may induce miR‐196a/b‐5p expression and suppress IGF2BP1 expression in certain gastric cancer contexts [43], which contrasted with a lot of studies [40, 41, 42, 44, 45, 46]. We fully respect the diversity of the mechanisms underlying the development of gastric cancer. Different basal expression patterns, genetic backgrounds, and stress‐response signaling in these lines may lead to divergent regulatory outcomes. Thus, further research is necessary to confirm the role of IGF2BP1 in *H. pylori‐*associated GC.

Ferroptosis is a distinct form of regulated cell death characterized by iron‐dependent lipid peroxidation and is genetically and mechanistically distinct from apoptosis and necroptosis [47]. Emerging evidence highlights epitranscriptomic regulation as an important layer of ferroptosis control in cancer [48, 49]. METTL3‐mediated m^6^A modification of SLC7A11 has been shown to confer ferroptosis resistance in hepatoblastoma [50]. Consistent with these findings, our data support a model in which IGF2BP1 stabilizes m^6^A‐modified SLC7A11 transcripts to suppress ferroptosis in gastric epithelial cells, suggesting that epitranscriptomic control of ferroptosis may represent a conserved oncogenic strategy across tumor types. Interestingly, accumulating evidence has demonstrated that *H. pylori* or *H. felis* infection can significantly disrupt systemic and local iron homeostasis [51, 52]. Infection is associated with altered gastric iron handling, increased iron sequestration within the gastric mucosa, and inflammatory responses that contribute to systemic iron deficiency, as reported in previous studies [53, 54, 55]. Importantly, these findings suggest a compartmentalized iron paradox during chronic *Helicobacter* infection, characterized by local disturbances in gastric iron metabolism alongside systemic iron depletion. Within the gastric microenvironment, dysregulated iron handling may promote oxidative stress and lipid peroxidation, thereby creating conditions that are permissive for ferroptosis during early stages of infection. However, during chronic infection and disease progression, epithelial cells may undergo adaptive reprogramming to counteract sustained oxidative and ferroptosis stress. In this context, our findings suggest that *H. pylori*‐induced upregulation of IGF2BP1 contributes to ferroptosis resistance by stabilizing SLC7A11 expression, thereby enhancing cystine uptake and antioxidant capacity. This adaptive response may enable infected epithelial cells to survive under conditions of persistent inflammatory and iron‐associated stress. Collectively, these observations support a model in which *Helicobacter*‐associated iron dysregulation initially promotes oxidative stress and ferroptosis pressure, while subsequent activation of the IGF2BP1‐SLC7A11 axis facilitates epithelial survival and contributes to disease progression and malignant transformation.

From a therapeutic perspective, IGF2BP1 has emerged as an attractive but challenging target. Several pharmacological strategies targeting IGF2BP1 have been reported, including small‐molecule inhibitors, antisense oligonucleotides, and indirect approaches targeting IGF2BP1 phosphorylation or stability [30, 31, 56, 57, 58, 59, 60]. In our study, pharmacological disruption of IGF2BP1‐RNA interactions using BTYNB suppressed tumor growth in xenograft models, particularly in tumors with high IGF2BP1 expression, and was associated with enhanced lipid peroxidation and ferroptosis. Importantly, we further demonstrate that Cucurbitacin B exerts similar inhibitory effects on IGF2BP1‐SLC7A11 signaling and suppresses gastric cancer growth in patient‐derived organoids, suggesting that independent chemical scaffolds can converge on IGF2BP1 inhibition. The consistent inhibitory effects observed in these ex vivo models support the translational potential of targeting IGF2BP1‐dependent pathways in gastric cancer.

In summary, our study identifies IGF2BP1 as a key mediator of *H. pylori*‐associated gastric carcinogenesis by linking infection‐induced epitranscriptomic reprogramming to ferroptosis resistance. By integrating cellular, animal, and patient‐derived models, we highlight IGF2BP1 as both a mechanistic driver and a potential therapeutic target in gastric cancers arising in the context of chronic *H. pylori* infection.

## 4 Experimental Section/Methods

### 4.1 *H. pylori* Strains

*H. pylori* strains ATCC 26695, PMSS1, and *Helicobacter felis* (*H. felis*) were used in this study as described previously [61]. The *cagA* and *vacA* knockout strains were constructed by our laboratory and verified via PCR and sequencing following the established protocols [10]. The strains were cultured on blood agar plates containing 39 g/L Columbia solid culture medium (Oxoid), 5% (v/v) sheep blood (Curtin Matheson, Jessup, MD, USA) and supplemented with *Helicobacter pylori* selective supplement (DENT) (Oxoid, SR0147E) in a microaerobic environment [5% (v/v) O_2_, 10% (v/v) CO_2_ and 85% (v/v) N_2_] at 37°C. Additionally, the knockout strains were subjected to antibiotic selection during cultivation. *H. pylori* were directly harvested from 24‐ to 48‐h plate cultures. Before harvesting, all strains underwent examination via Gram staining, urease tests, oxidase tests, and catalase tests to confirm their identity. All cell experiments were carried out using *H. pylori* strain 26695 in co‐culture. *H. pylori s*train PMSS1 was used in the conventional mouse model, while strain *H. felis* was used in the SPEM mouse model.

### 4.2 Cell Culture and Co‐Culture With *H. pylori*

Human gastric epithelial GES‐1 cells, along with gastric cancer cell lines BGC823 and SGC7901, were cultured in Roswell Park Memorial Institute (RPMI) 1640 medium supplemented with 10% (v/v) fetal bovine serum (FBS) (PAN‐Biotech, Adenbach, Germany) at 37°C in a humidified incubator at 5% (v/v) CO_2_. Cell line authentication via STR analysis and routine testing for mycoplasma infection was performed. The IGF2BP1 overexpression and knockout stable cell lines were selected with puromycin (Life Technologies).

For in vitro co‐culture with gastric epithelial cells and gastric cancer cells, *H. pylori* ATCC 26695 and knockout strains were utilized at a multiplicity of infection (MOI) of 100:1, following established procedures [62]. When the cells were harvested, the free *H. pylori* was washed three times with cold PBS, after which the cells were centrifuged to collect. SR11302 (HY‐15870, MCE, New Jersey, USA) was used to inhibit AP‐1 with a final concentration of 2 µm.

### 4.3 Patient Samples and Organoid Culture

The human gastric mucosa samples were collected from patients undergoing gastroendoscopy at Peking University Third Hospital (Beijing, China), and Warthin‐Starry staining was used to confirm whether *H. pylori* was present.

Patient‐derived organoids (PDOs) were cultured and passaged according to our established standard protocols [63]. Briefly, tissue samples were rinsed with D‐PBS (Solarbio, D1040) containing 100 U/mL of penicillin‐streptomycin‐amphotericin B, cut into sections, and digested with an enzymolysis reagent consisting of advanced DMEM/F12, type I (Sigma–Aldrich, V900891‐100MG), type II (Gibco, 17101015), and type IV collagenases (Worthington, LS004188) at 37°C. The tissues were periodically disrupted every 5 min until no visible cell mass remained. The solution was then filtered through a 70‐µm cell mesh and centrifuged at 300 g for 5 min. The cells were then suspended in a gastric organoid medium (Kingculture Organoid Growth Medium, KCW‐1) mixed with Matrigel (R&D, 3536‐010‐02) in a 1:1 ratio and incubated in preheated 24‐well plates at 37°C under a 5% CO_2_ atmosphere. After 30 min, when the Matrigel solidified, 600 µL of gastric organoid medium was added to each well and replaced every 3 days. For passaging, organoids were collected by disrupting the Matrigel droplets. Following centrifugation and resting, 1 mL of preheated TrypLE (Gibco, 12604021) was added for organoid digestion and dissociation. The enzymolysis process was closely monitored until the organoids dissociated into individual cells, at which point digestion was stopped. The cells were then cultured as previously described, and organoids were passaged weekly.

These studies were approved by the Medical Ethics Committee of the Peking University Third Hospital institutional review board, and written informed consent was obtained from all patients (Approval No.: M2019007 and IRB00006761‐M2021434).

### 4.4 Mouse Models

Six‐week‐old male specific pathogen‐free (SPF) C57BL/6 mice were obtained from the Laboratory Animal Science of Peking University (Beijing, China). The mice were divided into two groups. Group 1 served as the negative control (NC) group and received Brucella broth alone via intubation. Group 2 was the *H. pylori*‐infected group, in which the PMSS1 strain or *H. felis* was utilized to infect gastric epithelial cells of C57BL/6 mice by oral gavage every other day for a total of five administrations, with each administration consisting of 0.5 mL Brucella broth containing 3 × 10^8^ colony‐forming units (CFUs) of *H. pylori* PMSS1.

To determine whether elevated IGF2BP1 is sufficient to drive tumor growth, ferroptosis resistance, as well as the anti‐tumor efficacy of the small‐molecule inhibitor targeting IGF2BP1 in vivo, a tumor xenograft formation model was constructed. BGC823‐pCDH and BGC823‐IGF2BP1 cells (3 × 10^6^ cells in 150 µL PBS) were subcutaneously injected into the left and right flanks of 12 five‐week‐old BALB/c male nude mice, respectively. Subsequently, these mice were randomly allocated into two groups. One group (12 mice) received daily intratumoral injections of 50 µL BTYNB (10 mm), while the other group (12 mice) received daily intratumoral injections of solvent control, phosphate‐buffered solution (PBS) for 3 weeks. The tumor blocks were collected when the mice were sacrificed under anesthesia at 3 weeks post‐injection. Tumor xenografts were measured twice per week for 6 weeks, and tumor volume was calculated following the formula [2]:

$$\text{tumor volume}=0.52\times \text{length}\times {\text{width}}^{2}$$

The animal research was authorized by the Ethics Committee of the Peking University Third Hospital (Approval No. SA2022211).

### 4.5 RNA Extraction and Quantitative PCR Analysis (RT‐qPCR)

Total RNA was extracted from cells or tissues using TRIzol reagent (Invitrogen, Carlsbad, CA) according to the manufacturer's protocol. The total RNA (2 µg) of every sample was reverse transcribed into cDNA using Fast‐King RT Kit and qPCR Mix SYBR Green Kit (Tiangen Biotech, Beijing, China) in accordance with the manufacturer's instructions. The iQ5 real‐time PCR detection system (Bio‐Rad Laboratories) was used with the Fast‐King qPCR Mix SYBR Green Kit (Tiangen Biotech) according to the manufacturer's instructions. Relative expression or copy numbers of each target gene were determined by normalization to the expression of *ACTB* (for human gene) and *Actb* (for mouse gene). The primers used for RT‐qPCR are provided in Table S2.

### 4.6 Transfection and Construction of Stable Overexpressing or Knockout Cell Lines

Plasmid transfections in the cells were performed using Lipofectamine 2000 (Invitrogen). Furthermore, the introduction of siRNA targeting candidate genes or siRNA control (siNC) was accomplished via Lipofectamine RNAiMAX Transfection Reagent (Invitrogen) following the manufacturer's protocol. To construct IGF2BP1 overexpression or knockout cells, HEK 293T cells were transfected with a lentivirus plasmid. The lentiviruses were produced by HEK 293T cells, and the cells were infected with the lentivirus. The stable cell lines were screened out by puromycin (Yuanye, Shanghai, China). All siRNAs we used in the experiment are shown in Table S3, and the sgRNAs we used are shown in Table S4.

### 4.7 Western Blot

Cells were washed with cold PBS and then lysed with radioimmunoprecipitation buffer (Solarbio, Beijing, China) with protease and phosphatase inhibitors (Solarbio) on ice for 30 min. The concentration of total protein was quantified using the BCA Protein Assay kit (Thermo Fisher Scientific, Waltham, Massachusetts, USA). Equal amounts of protein were resolved by sodium dodecyl sulfate‐polyacrylamide gel electrophoresis and then transferred to polyvinylidene fluoride membranes. After blocking with 5% nonfat milk for 1 h at room temperature, the membranes were incubated overnight at 4°C with the primary antibodies. After three washes in TBST supplemented with 0.1% (v/v) Tween‐20, the membranes were then incubated with a secondary antibody, and the bands were scanned by an Odyssey Infrared Imaging System (LI‐COR Biosciences, Lincoln, NE, USA) or Tanon 5200 Fully Automated Chemiluminescence Image Analysis System (Tanon, Shanghai, China) with enhanced chemiluminescence reagent (Yamei, Shanghai, China). All antibodies we used in the experiment are shown in Table S5.

### 4.8 Histopathological Examination

For the mouse sample, mice were sacrificed at a specific time point, and their stomach tissues were collected and fixed overnight using 10% neutral formalin. Samples of patients and mice were used to prepare formalin‐fixed paraffin‐embedded tissue sections at 4 µm. The staining with hematoxylin and eosin was performed using the Modified Hematoxylin‐Eosin (HE) Stain Kit (G1121, Solarbio) following the manufacturer's protocol.

Immunohistochemistry was performed as described previously [64]. Briefly, the sections were dewaxed in xylene and rehydrated in a gradient of ethanol concentrations. Then, antigen retrieval was performed, and the sections were incubated with the primary antibody overnight at 4°C. The sections were washed with PBS and incubated with an IgG‐horseradish peroxidase polymer (ZSGB‐BIO, Beijing, China). Lastly, the diaminobenzidine substrate was used for color development. All pathology results were reviewed and evaluated by a senior gastroenterology pathologist. All antibodies we used in the experiment are shown in Table S5.

### 4.9 Immunofluorescence

The organoid immunofluorescence experiment was performed as previously described [65]. Briefly, transfer suspended organoids from each well of an ultra‐low attachment well plate into a 1.5 mL conical microcentrifuge tube and incubate organoids in paraformaldehyde fixative solution for 30 min at room temperature. Then the organoids were washed with PBS and incubated overnight at 4°C with specific primary antibodies. The following day, the organoids were washed in PBS and incubated with secondary antibodies for 4 h at room temperature. The organoids were then washed in PBS, stained with Hoechst 33342 (Beyotime, Beijing, China) for 10 min and visualized using the Zeiss confocal microscope (Oberkochen, Baden‐Württemberg, Germany) at 400× magnification to ensure that they have normal gastric organ characteristics and polarity. All antibodies we used in the experiment are shown in Table S5.

### 4.10 Multiple Fluorescence Staining

The multiplex fluorescence staining kit was purchased from Absin (abs50014, Absin, Shanghai, China). The main steps were as follows: paraffin sections were heated at 37°C overnight and dewaxed in xylene, gradient alcohol dehydration, 10% neutral formalin immersion for 10 min; the antigen was repaired in EDTA solution using microwave repair and cooled to room temperature. After 10 min of blocking, the antibody of TFF2 was incubated for 1 h at room temperature; the secondary antibody was incubated for 10 min, then incubated with fluorescent dye for 10 min. After the microwave repair was cooled to room temperature, the above steps were repeated again to complete the staining of MIST1, CDX2, Ki‐67, ATP4A, and finally incubated with DAPI for 5 min to stain the nucleus and anti‐fluorescence quencher seal. The expression was observed under the fluorescence microscope. All antibodies we used in the experiment are shown in Table S5.

### 4.11 ROS detection

Dilute the DCFH‐DA probe with serum‐free culture medium at a ratio of 1:1000 to achieve a final concentration of 10 µmol/L. After co‐culturing *H. pylori* with cells, remove the cell culture medium and add an appropriate volume of the diluted DCFH‐DA. Incubate at 37°C in a cell culture incubator for 20 min. Wash the cells three times with serum‐free cell culture medium to thoroughly remove any DCFH‐DA that has not entered the cells. After collecting the cells, detect them using a fluorescence microplate reader with an excitation wavelength of 488 nm and an emission wavelength of 525 nm or visualize them using the Zeiss confocal microscope.

### 4.12 CCK‐8 Assay

GES‐1, BGC823, and SGC7901 cells were seeded into 96‐well plates and cultured for 24, 48, 72, 96, and 120 h, respectively. Then, the cells were incubated with 10 µL of Cell Counting Kit‐8 (CCK‐8) reagent (CK04, Dojindo Laboratories, Kyushu Island, Japan) for 2 h. The absorbance at 450 nm was read using a Spectrophotometer.

### 4.13 CellTiter‐Glo 3D Cell Viability Assay

The activity of organoids derived from patient samples was assessed using the CellTiter‐Glo 3D Reagent kit (Promega, Madison, USA) according to the manufacturer's instructions. Briefly, add 100 µL of CellTiter‐Glo 3D Reagent to 100 µL of medium containing cells in each well. Vigorously mix the contents for 5 min to induce cell lysis. Allow the plate to incubate at room temperature for an additional 25 min to stabilize the luminescent signal, then record the luminescence.

### 4.14 Cell Apoptosis Assay

Cell apoptosis was detected using the Annexin V, FITC Apoptosis Detection Kit (AD10, Dojindo Laboratories) according to the manufacturer's protocol. Briefly, after washing the cells twice with PBS, gently digest the cells with trypsin. Then transfer the cell suspension to a centrifuge tube, add the pre‐prepared 1×Annexin V Binding Solution to form a cell suspension with a final concentration of 1 × 10^6^ cells/mL. Take 100 µL of the cell suspension and add 5 µL of Annexin V, FITC conjugate to the cell suspension, then add 5 µL of PI Solution. Incubate at room temperature in the dark for 15 min and perform flow cytometry within 1 h.

### 4.15 Transmission Electron Microscope (TEM)

We used the transmission electron microscope to observe the mitochondrial morphology in cells. Cells cultured in a six‐well plate were harvested and fixed with a 5% glutaraldehyde solution for 24 h. After washing with PBS, the cells were treated with a dimethylsulfate buffer containing 0.1% tannic acid for 1 h, fixed with a 1% osmium tetroxide buffer at 4°C, and stained with a 1% uranyl acetate solution. After gradient dehydration and embedding, the samples were incubated at 45°C for 24 h before ultrathin sectioning. Digital images were obtained using the high‐resolution field emission transmission electron microscope (HRTEM‐JEM‐F200, JEOL, Japan).

### 4.16 Lipid Peroxidation (LPO) Detection

The LPO was detected using the Lipid Peroxidation Probe‐BDP 581/591 C11 (L267, Dojindo Laboratories). After removing the culture medium and washing the cells twice with culture medium, the cells were resuspended with the BDP 581/591 C11 working solution and incubated at 37°C in a 5% CO_2_ incubator for 30 min. Then the cells were washed twice with HBSS, and HBSS was added again for flow cytometry analysis.

### 4.17 Dot Blot

Extract RNA samples and measure RNA concentration, then dilute the sample to 500 ng/µL in a new RNase‐free EP tube and mix thoroughly. After centrifugation, heat the RNA sample at 95°C for 3 min to denature the secondary structure, and then immediately place it on ice to cool, preventing the reformation of secondary structure. Use the blunt end of a 1 mL pipette tip to mark the NC membrane for sample loading guidance. Trim the NC membrane to an appropriate size and transfer it to a clean plastic container. Dispense 2 µL of RNA sample onto the NC membrane in sequence. After the sample on the NC membrane dries slightly at room temperature, perform UV cross‐linking of RNA to the membrane under a UV lamp at 254 nm for 30 min. Then wash the membrane with 10 mL TBST for 5 min to remove unbound RNA. Add 10 mL blocking solution, incubate at room temperature for 1 h, and then add blocking solution containing m6A antibody and incubate at 4°C overnight. After three washes in TBST, incubate with the secondary antibody at room temperature for 1 h. The dots were scanned by Tanon 5200 Fully Automated Chemiluminescence Image Analysis System (Tanon, Shanghai, China) with ECL substrate. Then, place the membrane in 10 mL of methylene blue staining buffer and incubate at room temperature with gentle shaking for 30 min. After three washes with ddH_2_O until the background is mostly clear, capture an image of the methylene blue‐stained membrane under white light as a control.

### 4.18 RNA Immunoprecipitation (RIP)

RIP assays were performed as previously described [66]. In brief, GES‐1 cells were harvested, and the RIP lysate supernatants were prepared. The cell lysates were incubated with IGF2BP1 antibody (Proteintech) or rabbit IgG (Proteintech) embedded ProteinG beads at 4°C overnight. After extensive washing six times, the beads were resuspended using NT2 buffer with RNase‐free DNase I (Roche) and proteinase K (Roche). The coprecipitated RNAs were isolated by using phenol‐chloroform extraction and ethanol precipitation, and detected by quantitative real‐time PCR, and the products were visualized with ethidium bromide after electrophoresis in 1% agarose.

### 4.19 RNA Turnover Assay

To assess the half‐life of endogenous *SLC7A11* mRNA, we employed an RNA turnover assay as previously described [67]. The stable cells were treated with actinomycin D (MedChemExpress) at a concentration of 5 µg/mL. Then, at 0, 3, 6, 9, and 12 h post‐treatment, RNA was prepared using TRIzol and the level of remaining *SLC7A11* mRNA transcripts was determined by RT‐qPCR.

### 4.20 Scratch Wound‐Healing Assay

The IGF2BP1 overexpression and knockdown of GES‐1 and BGC823 cells with the control cells were seeded into culture plates. When the cells had grown to almost 90% confluence, the artificial wounds were made with pipette tips. The images of cell migration were captured at 0 and 48 h, and the ratio of migration was calculated using ImageJ.

### 4.21 RNA Transcriptome Analysis

Total RNA was extracted from cells according to the standard method, and three biological repeats were performed for each group. The RNA were assessed using the RNA Nano 6000 Assay Kit of the Bioanalyzer 2100 system (Agilent Technologies, CA, USA) and then prepared to structure the library, which was initially quantified by Qubit2.0 Fluorometer (Invitrogen, California, USA) for transcriptome sequencing, and the insert size of the library was detected using an Agilent 2100 bioanalyzer (Shanghai Asiagene Technology Co., Ltd, Shanghai, China) at Novogene Bioinformatics Technology Co., Ltd. Differential expression analysis was performed using the DESeq2 R package (1.20.0), which provides statistical routines to determine differential expression in digital gene expression data using a model based on the negative binomial distribution. The resulting *P*‐values were adjusted using Benjamini and Hochberg's approach to control the false discovery rate. *P*adj < 0.05 and |log2(fold change) | > 1 were set as thresholds for significantly differential expression. We used the clusterProfiler R package (3.8.1) to test the statistical enrichment of differentially expressed genes in the KEGG pathway. Reactome pathways with corrected *p*‐values less than 0.05 were considered significantly enriched by differentially expressed genes.

### 4.22 Statistical Analysis

Statistical analyses were performed using GraphPad Prism 8.0 software (GraphPad Software, San Diego, California, USA). All data were presented as mean ± standard error of the mean (SEM), and the differences between two independent groups were analyzed using a two‐tailed Student's *t*‐test. For multiple comparisons, one‐way ANOVA was used, followed by the Student‐Newman–Keuls test. *p*‐values < 0.05 were considered statistically significant.

## Author Contributions

**Jing Ning**: conceptualization, methodology, data curation, investigation, Writing – original draft, Writing – review and editing, visualization, funding acquisition, validation, software, formal analysis. **Xin Guan**: data curation, investigation. **Xinyu Hao**: methodology. **Jing Zhang**: methodology. **Deyao Li**: investigation, data curation, validation. **Ying Xiong**: investigation. **Wenlin Zhang**: investigation, resources. **Xiurui Han**: investigation. **Yanfei Lang**: investigation. **Meiling Zhou**: investigation. **Mengjie Yang**: investigation. **Zhi Huang**: methodology, visualization, software. **Tinghui Qu**: investigation, validation. **Ming Zu**: resources, investigation. **Qiao Meng**: investigation. **Weiwei Fu**: project administration. **Fengmin Lu**: methodology, conceptualization, project administration. Jing Zhang: writing – review and editing, project administration, resources. **Xiangmei Chen**: conceptualization, writing – review and editing, supervision, funding acquisition. **Tong Liu**: writing – review and editing, methodology, visualization. **Shigang Ding**: funding acquisition, project administration, resources, supervision, writing – review and editing.

## Funding

This work was supported by the National Science Foundation of China grant (Grant NO. 82270592, 82503993, and 82572552).

## Conflicts of Interest

The authors declare no conflicts of interest.

## Supporting information




**Supporting File**: advs77677‐sup‐0001‐SuppMat.docx.

## Data Availability

The RNA sequencing datasets generated in this study have been deposited in the Gene Expression Omnibus (GEO) database under accession number GSE338493. The data that support the findings of this study are available from the corresponding author upon reasonable request.
