# Supplementary material for: Helicobacter pylori‐Induced Persistent IGF2BP1 Activation Promotes Ferroptosis Resistance in Gastric Tumorigenesis
Source: Adv Sci (Weinh). 2026 Sep 11:e77677. Online ahead of print. doi: 10.1002/advs.77677 (PMC13564185; doi:10.1002/advs.77677)
Supplement: Supplementary file 1 [file ADVS-9999-e77677-s001.docx]

Supporting Information

*Helicobacter pylori*-induced persistent IGF2BP1 activation promotes ferroptosis resistance in gastric tumorigenesis

Jing Ning, Xin Guan, Xinyu Hao, Jing Zhang, Deyao Li, Ying Xiong, Wenlin Zhang, Xiurui Han, Yanfei Lang, Meiling Zhou, Mengjie Yang, Zhi Huang, Tinghui Qu, Ming Zu, Qiao Meng, Weiwei Fu, Fengmin Lu^*^, Jing Zhang^*^, Xiangmei Chen^*^, Tong Liu^*^, Shigang Ding^*^

**The file includes:**

Figure S1 to S12

Table S1 to S5

**Supplementary figures**

**Figure S1. Validation of successful *H. pylori* infection in the gastric epithelial cell model and mouse model.**

**(A)** Gastric epithelial cell line GES-1 was co-cultured with *H. pylori* 26695 at the multiplicity of infection (MOI) of 100:1. Immunofluorescence imaging demonstrated the presence of *H. pylori* (green) attached to gastric epithelial cells. **(B)** Western blot analysis further confirmed infection by detecting *the H. pylori* CagA protein in co-cultured cells, indicating effective bacterial-host cell interaction across all time points. **(C)** Representative *H. pylori* IHC staining images of the *H. pylori* PMSS1-infection group and the control group mice, which validated the successful bacterial infection *in vivo*. Scale bars, 100 μm.

**Figure S2. The expression level of IGF2BP1 was significantly upregulated after *H. pylori* infection.**

**(A)** and **(B)** The RT-qPCR results showed a significant upregulation of IGF2BP1 following *H. pylori* infection at 6, 12, 24, and 48 h in SGC7901 cells (A) and BGC823 cells (B). **(C)** and **(D)** Western blot analysis verified that IGF2BP1 protein levels were gradually upregulated as *H. pylori* infection duration increased in SGC7901 cells (C) and BGC823 cells (D). **(E)** LPS did not upregulate IGF2BP1 expression in GES-1 cells. **(F)** In the BGC823 and SGC7901 cells, the same results also showed that IGF2BP1 expression remained unchanged following LPS stimulation. **(G)** and **(H)** The western blot results showed that co-culture with *cagA-* (G) and *vacA-*deficient (H) strains still resulted in a significant upregulation of IGF2BP1. **(I)** The RT-qPCR results showed that IGF2BP1 mRNA levels were significantly upregulated in GES-1 cells co-cultured with *H. pylori* 26695. The AP-1 inhibitor (SR11302) significantly attenuated *H. pylori*-induced upregulation of IGF2BP1 in GES-1 cells. **(J)** The western blot analysis further demonstrated that AP-1 inhibition suppressed *H. pylori*-induced IGF2BP1 protein upregulation. *, *P*<0.05; **, *P*<0.01.

**Figure S3.** **The *H. felis* gavage mouse model recapitulates gastric mucosal disease progression.**

**(A)** The mouse model was established via *H. felis* gavage (n=6 per group) to simulate the progression of gastric mucosal disease. Histological analysis using HE staining (up) and multiplex fluorescence staining (bottom) revealed marked inflammatory infiltration at 2 months post-gavage, while the SPEM phenotype was observed at 6 months post-gavage.

**Figure S4. The basal expression levels of IGF2BP1 across the gastric cell lines.**

**(A)** The western blot result showed the different basal expression levels of IGF2BP1 in GES-1, AGS, BGC823, MKN45, and SGC7901 cell lines.

**Figure S5. IGF2BP1 overexpression suppresses erastin-induced cell death in gastric cancer cells.**

**(A)** In BGC823 and SGC7901 cells, IGF2BP1 overexpression significantly suppressed erastin-induced cell death, and in contrast, IGF2BP1 knockout significantly promoted cell death.

**Figure S6. The deficiency of IGF2BP1 promotes ferroptosis in gastric cancer cells.**

**(A)**-**(C)** Flow cytometry analysis of lipid peroxidation showed that knockout of IGF2BP1 enhanced erastin-induced lipid peroxidation in gastric cancer cells BGC823 (A) and SGC7901 (B). Following IGF2BP1 knockout, Fer-1 failed to rescue erastin-induced ferroptosis in both gastric cancer cell lines. The quantitative analyses are shown in (C). *, *P*<0.05; **, *P*<0.01.

**Figure S7. Transcriptomic profiling of IGF2BP1 overexpression and *H. pylori*-treated gastric epithelial cells.**

**(A)** The volcano plot of the RNA-sequencing dataset showing differentially expressed genes (DEGs) in the stable cell line with IGF2BP1 overexpression (n=3) and compared with control cells (n=3) (*P*< 0.05, fold-change [FC]≥2). **(B)** The heatmap of DEGs in two groups. **(C)** The volcano plot of the RNA-sequencing dataset showing differentially expressed genes (DEGs) in the GES-1 cells treated with *H. pylori* (n=3) compared with the control group (n=3) (*P*< 0.05, fold-change [FC]≥2). **(D)** KEGG pathway enrichment analysis of DEGs identified in *H. pylori*-treated cells.

**Figure S8. The intersection analysis of two transcriptomic datasets reveals enrichment in inflammatory and cell surface-associated pathways.**

**(A)** Venn diagram showing 245 overlapping DEGs between two transcriptomic analyses (fold-change ≥ 2), including SLC7A11, CHAC1, and multiple genes associated with oxidative stress and inflammatory responses. **(B)** Gene Ontology (GO) enrichment analysis indicated that these DEGs were primarily localized at the cell surface. **(C)** KEGG pathway enrichment analysis showed that these DEGs primarily influenced functions related to inflammation, cytokine-cytokine receptor interaction, and the pathways in cancer.

**Figure S9. IGF2BP1 deficiency exacerbates erastin-induced downregulation of SLC7A11.**

**(A)** Western blot results showed that IGF2BP1 knockout significantly reduced the SLC7A11 level and further potentiated erastin-induced downregulation of SLC7A11 and its downstream target GPX4. Treatment with the ferroptosis inhibitor ferrostatin-1 (Fer-1) partially restored SLC7A11 expression; however, levels remained lower than those in control cells.

**Figure S10. IGF2BP1 can bind *SLC7A11* mRNA in an m^6^A-dependent manner.**

**(A)** Using the SRAMP database to predict potential m^6^A RNA methylation sites within the *SLC7A11* mRNA sequence. The results showed 10 candidate m^6^A methylation sites (up), two of which matched the predicted IGF2BP1 recognition motifs (bottom). **(B)** RNA immunoprecipitation (RIP) assays using anti-IGF2BP1 antibody confirmed the interaction between IGF2BP1 and *SLC7A11* mRNA (up); enrichment of SLC7A11 was quantified by RT-qPCR and validated by PCR with agarose gel electrophoresis (down). IgG served as a negative control. **(C)** Western blot results confirm the successful construction of the IGF2BP1 KH domain deletion mutants. **(D)** The IP of the IGF2BP1 group and all KH domain deletion mutant groups, followed by a Western blot performed with an anti-FLAG antibody (up). Following the RIP assay, the coprecipitated SLC7A11 mRNA was detected by RT-qPCR and subjected to agarose gel electrophoresis following amplification by PCR (down). **(E)** The level of intracellular m6A modification was analyzed by Dot blot. Methylene blue staining served as a loading control. **(F)** The IP of all groups, followed by Western blot, was performed with anti-IGF2BP1 antibody, and the lysate was treated with RNase. **(G)** RIP assays using SLC7A11 3’UTR wild-type and mutant constructs demonstrated that mutation of predicted m^6^A sites significantly reduced IGF2BP1 binding (up), as validated by RT-qPCR and PCR (down).

**Figure S11. Genetic depletion of SLC7A11 impairs key cellular functions in gastric epithelial cells.**

**(A)** Western blot results showed that SLC7A11 was significantly diminished after SLC7A11 knockout. **(B)** and **(C)** RT-qPCR (B) and western blot (C) results showed that SLC7A11 was significantly diminished after SLC7A11 knockdown with siSLC7A11-2 and siSLC7A11-3. **(D)** Western blot results demonstrated that IGF2BP1 overexpression promoted the expression of downstream effectors of the SLC7A11 pathway under *H. pylori* stimulation. **, *P*<0.01.

**Figure S12. CuB significantly inhibits the proliferation of IGF2BP1-overexpressed gastric cancer cells.**

**(A)** The results of the CCK8 assay indicated that, in the gastric cancer cell lines BGC823 and SGC7901, IGF2BP1 overexpression effectively promoted the proliferation of gastric cancer cells. Treatment with Cucurbitacin B (CuB) markedly inhibited cell proliferation and reversed the IGF2BP1-induced proliferative advantage. *, *P*<0.05; ***, *P*<0.001.

**Table S1. Basic characteristics of the patients in the cohort**

|  | ***H. pylori*-negative group (n=116)** | ***H. pylori*-positive group (n=116)** | ***P* value** |
| --- | --- | --- | --- |
| **Age (years)** | 48.00  (38.00, 58.00) | 48.50  (34.25, 59.75) | 0.78 |
| **Male/Female** | 56/60 | 63/53 | 0.64 |

**Table S2. Primer sequences used for RT-qPCR**

| **Primer** | **Sequence（5'-3'）** |
| --- | --- |
| ACTB qF (Homo) | TTGTTACAGGAAGTCCCTTGCC |
| ACTB qR (Homo) | ATGCTATCACCTCCCCTGTGTG |
| IGF2BP1 qF (Homo) | GGCCATCGAGAATTGTTGCAG |
| IGF2BP1 qR (Homo) | CCAGGGATCAGGTGAGACTG |
| *Igf2bp1* qF (Mus) | CGGCAACCTCAACGAGAGT |
| *Igf2bp1* qR (Mus) | GTAGCCGGATTTGACCAAGAA |
| *Actb* qF (Mus) | GGCTGTATTCCCCTCCATCG |
| *Actb* qR (mus) | CCAGTTGGTAACAATGCCATGT |
| SLC7A11 qF (Homo) | TCTCCAAAGGAGGTTACCTGC |
| SLC7A11 qR (Homo) | AGACTCCCCTCAGTAAAGTGAC |
| GPX4 qF (Homo) | GAGGCAAGACCGAAGTAAACTAC |
| GPX4 qR (Homo) | CCGAACTGGTTACACGGGAA |
| NFE2L2 qF (Homo) | TTCCCGGTCACATCGAGAG |
| NFE2L2 qR (Homo) | TCCTGTTGCATACCGTCTAAATC |
| TGFBR1 qF (Homo) | GCTGTATTGCAGACTTAGGACTG |
| TGFBR1 qR (Homo) | TTTTTGTTCCCACTCTGTGGTT |
| METTL3 qF (Homo) | CATTGCCCACTGATGCTGTG |
| METTL3 qR (Homo) | AGGCTTTCTACCCCATCTTGA |
| METTL14 qF (Homo) | GAACACAGAGCTTAAATCCCCA |
| METTL14 qR (Homo) | TGTCAGCTAAACCTACATCCCTG |
| HP 16srRNA qF | CCGCCTACGCGCTCTTTAC |
| HP 16srRNA qR | CTAACGAATAAGCACCGGCTAAC |

**Table S3. siRNA sequences used for gene knockdown**

| **siRNA** | **Sequence（5'-3'）** |
| --- | --- |
| siIGF2BP1-1 | GGCTCAGTATGGTACAGTA |
| siIGF2BP1-2 | TGAAGATCCTGGCCCATAA |
| siIGF2BP1-3 | GAAGGACGGAACCTGAAGA |
| siMETTL3-1 | CAAGTATGTTCACTATGAA |
| siMETTL3-2 | GACTGCTCTTTCCTTAATA |
| siMETTL3-3 | GGACTCGACTACAGTAGCT |
| siMETTL14-1 | CAACTACAATGCAGAAACA |
| siMETTL14-2 | GAAGACGCCTTCATCTATT |
| siMETTL14-3 | GGACCAACGCTTACAAATA |
| siSLC7A11-1 | GGAAGAGATTCAAGTATTA |
| siSLC7A11-2 | CTTGCAATATGTATATCCA |
| siSLC7A11-3 | GCTGCTGCTTTCAAATGCA |

**Table S4. sgRNA oligos sequences used for gene knockout**

| **sgRNA oligos** | **Sequence（5'-3'）** |
| --- | --- |
| CRISPR-IGF2BP1-gRNA1 UP | CACCGgagcgtgacccccgcggact |
| CRISPR-IGF2BP1-gRNA1 BOTTOM | AAACagtccgcgggggtcacgctcC |
| CRISPR-IGF2BP1-gRNA2 UP | CACCGgagcacaagatctcctacag |
| CRISPR-IGF2BP1-gRNA2 BOTTOM | AAACctgtaggagatcttgtgctcC |
| CRISPR-SLC7A11-gRNA1 UP | CACCGgttgtgtccaccatctccaa |
| CRISPR-SLC7A11-gRNA1 BOTTOM | AAACttggagatggtggacacaacC |
| CRISPR-SLC7A11-gRNA2 UP | CACCGtgggcaacaaggagccacct |
| CRISPR-SLC7A11-gRNA2 BOTTOM | AAACaggtggctccttgttgcccaC |

**Table S5. Detailed information about the antibodies used in this study**

| **Name** | **Vendor** | **Cat.** | **Application** |
| --- | --- | --- | --- |
| IGF2BP1 Rabbit Polyclonal antibody | Proteintech | 22803-1-AP | WB, RIP, IHC |
| SLC7A11/xCT Rabbit Polyclonal antibody | Proteintech | 26864-1-AP | WB, IHC |
| Rabbit monoclonal [EPNCIR144] anti-Glutathione Peroxidase 4 | abcam | ab125066 | WB, IHC |
| Rabbit polyclonal anti-4-Hydroxynonenal | abcam | ab46545 | IHC |
| *Helicobacter pylori* antibody (IHC) | ZSGB-BIO | ZA-0127 | IHC |
| CagA antibody (A-10) | Santa Cruz | sc-28368 | WB |
| MUC5AC Mouse Monoclonal Antibody (45M1) | Invitrogen | MA512178 | IF |
| β-Catenin (D10A8) XP® Rabbit mAb | CST | 8480 | IF |
| m^6^A Recombinant Rabbit Monoclonal Antibody | HUABIO | HA721152 | Dot Blot |
| METTL3 Rabbit Polyclonal antibody | Proteintech | 15073-1-AP | WB |
| METTL14 Rabbit Polyclonal antibody | Proteintech | 26158-1-AP | WB |
| Rabbit IgG control Polyclonal antibody | Proteintech | 30000-0-AP | RIP |
| Hoechst 33342 | Beyotime | C1027 | IF |
| Anti-Ki67 antibody | Abcam | ab15580 | Multiple IF |
| MIST1/bHLHa15 (D7N4B) XP® Rabbit mAb | CST | 14896 | Multiple IF |
| Anti-TFF2 antibody | Abcam | ab267474 | Multiple IF |
| Anti-Proton Pump (H, K-ATPase α subunit) mAb | MBL | D031-3 | Multiple IF |
| Anti-CDX2 antibody | Abcam | ab76541 | Multiple IF |
| goat anti-rabbit IRDye 800CW | LICOR | 926-32211 | WB |
| goat anti-mouse IRDye 680RD | LICOR | 926-68070 | WB |
| anti-rabbit IgG, HRP-linked antibody | CST | 7074 | WB |
| anti-mouse IgG, HRP-linked antibody | CST | 7076 | WB |
| β-Actin Mouse mAb | Abclonal | AC004 | WB |
